# Accumulation of damaged mitochondria in alveolar macrophages with reduced OXPHOS related gene expression in IPF

**DOI:** 10.1186/s12931-019-1196-6

**Published:** 2019-11-27

**Authors:** Eliza Tsitoura, Eirini Vasarmidi, Eleni Bibaki, Athina Trachalaki, Chara Koutoulaki, George Papastratigakis, Sevasti Papadogiorgaki, George Chalepakis, Nikos Tzanakis, Katerina M. Antoniou

**Affiliations:** 1 Department of Respiratory Medicine, Laboratory of Molecular and Cellular Pneumonology, Medical School, University of Crete, Heraklion, Crete Greece; 2 Department of Respiratory Medicine, University Hospital of Heraklion, Heraklion, Greece; 3 Electron Microscopy Laboratory, University of Crete, Heraklion, Greece

**Keywords:** Idiopathic pulmonary fibrosis, Mitochondria, Mitophagy, Autophagy, Alveolar macrophages

## Abstract

**Background:**

Impaired mitochondria homeostasis and function are established hallmarks of aging and increasing evidence suggests a link with lung fibrosis. Mitochondria homeostasis may be also affected in alveolar macrophages (AMs) in idiopathic pulmonary fibrosis (IPF). In this study, we used bronchoalveolar lavage (BAL), a tool for both clinical and research purposes, and a rich source of AMs.

**Methods:**

BAL samples were examined from 52 patients with IPF and 19 healthy individuals. Measurements of mitochondria reactive oxygen species (mtROS), mitochondria morphology and related gene expression were performed. Additionally, autophagy and mitophagy levels were analysed.

**Results:**

Mitochondria in AMs from IPF patients had prominent morphological defects and impaired transcription paralleled to a significant reduction of mitochondria homeostasis regulators PINK1, PARK2 and NRF1. mtROS, was significantly higher in IPF and associated with reduced expression of mitochondria-encoded oxidative phosphorylation (OXPHOS) genes. Age and decline in lung function correlated with higher mtROS levels. Augmentation of damaged, oxidised mitochondria in IPF AMs however was not coupled to increased macroautophagy and mitophagy, central processes in the maintenance of healthy mitochondria levels.

**Conclusion:**

Our results suggest a perturbation of mitochondria homeostasis in alveolar macrophages in IPF.

## Background

Idiopathic Pulmonary Fibrosis (IPF) is a chronic lung disease, characterized by poor prognosis, primarily affecting older individuals [1]. IPF pathogenesis is attributed to an accelerated apoptosis of Type-II pneumocytes coupled to an aberrant wound healing process, involving defective re-epithelization, abnormal fibroblast/myofibroblast apoptosis and excessive accumulation of extracellular matrix components [2]. Age associated changes, including telomere attrition, defective autophagy, mitochondrial dysfunction and cell senescence, are linked to IPF pathogenesis [3].

Mitochondria drive ATP production through oxidative phosphorylation (OXPHOS) and they represent the major generator of reactive oxygen species (ROS). In the last decade, it has become evident that cellular metabolism is intertwined with immune responses while, mitochondria activity largely shapes immune cell functions [4]. Mitochondrial function declines with age, resulting in morphological alterations, loss of cristae, reduced mitochondria respiratory chain (MRC) function and increased ROS production which is associated with mtDNA damage, fueling a vicious cycle of mitochondria impairment [5]. Mitochondrial homeostasis is essential for cellular health, and is tightly regulated by biogenesis and degradation/recycling [6]. PTEN-induced kinase 1 (PINK1) and E3 ubiquitin ligase (Parkin - encoded by PARK2 gene) represent key effector proteins of mitophagy, a selective form of macroautophagy, participating in the clearance of oxidized and damaged mitochondria [7].

Accumulation of damaged mitochondria is associated with IPF and the aging lung [3, 8, 9]. PINK1 deficiency and insufficient mitophagy in alveolar epithelial cells leads to increased mitochondrial damage and progression of fibrosis [10]. Conversely, PINK1 upregulation in mice contributes to elimination of fibrosis [11]. PINK1 decrease in fibrotic foci in IPF, and TGF-β treatment of lung fibroblasts lowers PINK1 and increases ROS levels [12]. PARK2 knockdown induces mitochondrial damage, increases mtROS production and promotes fibrosis through enhancement of apoptotic resistant phenotype of fibroblasts and myofibroblast differentiation [13]. MtROS is linked to mtDNA damage and evidence from a recent study in IPF showed a higher mutation rate in mitochondrial tRNA, probably affecting intra-mitochondrial translation [14]. Importantly, a widely used antidiabetic medication, metformin, ameliorated lung fibrosis in mice through beneficial effects on mitochondria, highlighting their pathogenetic role in pulmonary fibrosis [15].

Lung macrophages are a fluctuating and diverse population with a central role in inflammation, host defense, and tissue homeostasis [16]. It is well documented that macrophages within the lung orchestrate the downstream progression and maintenance of fibrosis [17]. A substantial expansion of macrophages in distal lung tissue is observed in IPF and a profibrotic macrophage population that appears in the lung during the development of fibrosis is currently under investigation [18, 19]. ROS production is required for the alternative activation of macrophages and can be attenuated by ROS inhibitors including N-acetyl-cysteine [20]. Interestingly, generation of mtROS from AMs has been causatively related to the development of asbestos-induced pulmonary fibrosis [21]. Mitochondria homeostasis in IPF AMs is not well characterized and in this study we sought to evaluate mitochondrial ROS levels, morphology, and mitophagy in AMs isolated from BAL in IPF patients.

## Materials and methods

### Patients

Seventy one (71) patients were prospectively enrolled in the Department of Thoracic Medicine, University Hospital of Heraklion, Crete, Greece from 2014 to 2018. Fifty two [52] patients with IPF and nineteen [19] healthy controls underwent bronchoscopy and Bronchoalveolar Lavage Fluid (BAL) was obtained.

IPF group: The diagnosis of IPF was based on ATS/ERS criteria or on multidisciplinary discussion [22]. Bronchoscopy was performed in treatment naive IPF patients as a part of the diagnostic approach.

All patients were evaluated within 1 month from bronchoscopy, with complete pulmonary function tests (PFTs). Lung volumes (forced expiratory volume in 1 sec – FEV_1_, forced vital capacity – FVC), using the helium-dilution technique and diffusion capacity (DLco, corrected for haemoglobin) using the single breath technique. The computerized system (Jaeger 2.12; MasterLab, Würzburg, Germany) was used and predicted values were obtained from the standardized lung function testing of the European Coal and Steel Community, Luxembourg (1993).

Control group: Control subjects were patients undergoing bronchoscopy for the investigation of haemoptysis and BAL sampling was performed at least 6 weeks following hospitalization. No signs of infection, ILD abnormalities or putative malignant lesions in the CT scans were observed. Furthermore, during bronchoscopy, no malignant lesions in the airways were observed, or abnormal mucus secretion was noted. BAL cellularity and differential BAL cell count were within the normal range. Additionally, BAL samples testing positive for bacteria growth were excluded. Since controls were healthy subjects no PFTs were performed.

Patients were classified as non-smokers, current smokers or former smokers (defined as having smoked a minimum of one cigarette a day for a minimum of 1 year, stopping at least 6 months before presentation).

Demographic data, pulmonary function tests, and BAL differential cell count are summarised in Table 1.

**Table 1:** Clinicopathological characteristics of all subjects included in the study. Values are expressed as median with range

Characteristics	Normal	IPF
Number	19	52
Gender (Male/Female)	10/9	39/13
Packyears	30(0–100)	26(0–150)
Non smokers	1	18
Former smokers	3	27
Current smokers	15	7
Age (years)	62(40–74)	74(56–84)
Macrophages	91(74–99)	87.25(49–97)
Lymphocytes	5(0.5–25)	5.37(0.25–45)
Neutrophils	1(0–8.8)	4.75(0.25–45)
Eosinophils	0(0–1.25)	1(0–7)
FVC		85 ± 20
FEV1		93 ± 18
FEV1/FVC		85.5 ± 5.8
TLC		76 ± 14
DLco		57 ± 19.5
Kco		87 ± 20

### BAL cell isolation and determination of cellular composition

Bronchoalveolar Lavage (BAL) was obtained as previously reported [23]. In brief, BAL was obtained from all patients at room temperature. A flexible bronchoscope was wedged into a sub-segmental bronchus of a predetermined region of interest based on radiographical findings. A BAL technique was performed by instilling a total of 180 ml of normal saline in 60-mL aliquots, each retrieved by low suction.

The BAL samples were subsequently kept on ice and were processed within 2 h of collection. Samples were filtered through sterile 70 nm cell strainers (BD) and centrifuged at 1500 rpm for 5 min at 4^ο^C. Cell pellets were washed and re-suspended with cold PBS. Total cell count and cell viability were subsequently assessed using Trypan blue (ICN). Differential cell population count was analysed following May-Grunewald-Giemsa staining as previously described [24].

### BAL cell isolation and AM culture

In order to study alveolar macrophages, by immune fluorescence or western blot, we enriched their population by pre-culture on glass slides or plastic dishes respectively, followed by washing of non-adherent cells such as lymphocytes, eosinophils and neutrophils, minor components of the BAL cell population. The remaining attached cell population comprises of macrophages and monocytes from the alveolar space. 0.5 million freshly isolated BAL cells were cultured in 24 well plates in DMEM (Biosera) growth media supplemented with 2% FCS (Biosera) and penicillin-streptomycin in a humidified incubator at 37 °C containing 5% CO_2_ for 1 h, with subsequent washes to remove non-adherent cells, allowing enrichment for alveolar macrophages/ monocytes.

### BAL cell surface marker expression analysis

0.5 million freshly isolated BAL cells were resuspended in PBS, 2% FCS and 2 mM EDTA buffer and labelled with pan-leucocyte marker CD45-FITC (#IQP-124F, IQProducts), macrophage/DC marker CD11c-PE-Cy5 (#301610, Biolegend), or CD45-FITC, monocyte marker CD14-PE-Cy5 (#A07765, IOTest, Beckman Coulter). CD163-PE-Cy7 (#333614, Biolegend) and appropriate Isotype controls. Data were acquired with Beckman Coulter flow cytometer and analyzed with FlowJo 8.7 (Treestar, Ashland, OR).

### Mitochondrial ROS measurement

Mitochondrial ROS was measured by MitoSOX™Red (Invitrogen) staining. MitoSOX™red is targeted to mitochondria in live cells and is readily oxidized by superoxide reactive oxygen species. Initially, the staining of oxidized mitochondria with MitoSOX following treatment with hydrogen peroxide (H_2_O_2_) was assessed. A549 cells, a non-small cell lung cancer (NSCLC)-derived human alveolar epithelial cell line, were treated with 1000 μM H_2_O_2_ and the characteristic staining for the mitochondrial network in the cytoplasm was observed which was further enhanced upon H_2_0_2_ treatment and mitochondria oxidation (Additional file 1: Figure S1a). Secondly, flow cytometry analysis of MitoSOX staining was tested on of THP1 human monocytes. THP1 cells showed a strong fluorescent MitoSOX signal (Additional file 1: Figure S1b) which was greatly enhanced following 1000 μM H_2_O_2_ treatment. Necrotic and apoptotic cells were expected to contain oxidized mitochondria and since H_2_O_2_ treatment is known to induce apoptosis, thus Propidium Iodide (PI) staining was analysed in parallel which allowed for the exclusion of the MitoSOX positive necrotic/apoptotic population from the analysis of the alive MitoSOX positive cells (Additional file 1: Figure S1b). A high proportion of MitoSOX positive cells upon H_2_O_2_ treatment was present despite the exclusion of the PI negative cell population (Additional file 1: Figure S1b) and the same PI positive population exclusion strategy was used subsequently in human BAL cells.

0.5 million freshly isolated BAL cells were cells were resuspended in RPMI-1640 supplemented with 2% FCS were stained with MitoSOX Red at a final concentration of 5 μM and CD45-FITC for 10 min at 37 °C. For FL-2, MitoSOX staining quantification, alveolar macrophages populations was selected according to high side scatter SSC^high^ and CD45^+^(Additional file 1: Figure S1c and d). Propidium Iodide (PI) was added to cells not previously stained with MitoSOX at a final concentration of 1 ng/ml, for 5 min immediately before flow cytometry analysis, for the detection of necrotic/apoptotic cells. A CD45-FITC stained control for each patient sample served as the unstained control sample for subsequent quantifications. PI staining allowed the exclusion of apoptotic/necrotic cells. Despite the PI exclusion gating strategy, a small percentage of Propidium Iodide (PI) positive cells was still among the MitoSOX positive analysed population and it was subtracted from the MitoSOX positive cell percentage during quantifications shown in Fig. 2b.

The percentage of MitoSOX positive cells was determined by the percent of cells showing FL-2 fluorescence higher than the unstained control, followed by subtraction of the FL-2 PI positive percentage of cells. Relative mean fluorescence intensity (MFI) was calculated by normalizing the MFI of the FL-2 channel/MitoSOX positive cells by the MFI of the FL-2 channel of the unstained cells since patient samples displayed wide ranges of autofluorescence. Data were acquired with Beckman Coulter flow cytometer and analyzed with FlowJo 8.7 (Treestar, Ashland, OR).

### RNA extraction and mRNA expression

1–1.5 million cells were centrifuged and cell pellets were homogenised in TriReagent™(MBL) for total RNA, followed by storage at -80^ο^C. Total RNA extraction and cDNA synthesis were performed as previously described [24]. Probe and primer sequences are summarized in Additional file 2: Table S1. GAPDH levels were used as endogenous control for the normalization of mRNA expression levels in BAL samples. Gene expression analysis was performed following incorporation of relative expression values in average (duplicates) normalized by GAPDH. Relative expression values for the patient cohort were calculated by 2-^ΔΔCt^ method, where ΔΔCt = (sample Ct GOI-sample Ct GAPDH)-(Calibrator Ct GOI- Calibrator Ct GAPDH), GOI = gene of interest, calibrator = mean of all Cts.

### mtDNA/ gDNA ratio determination

Total DNA isolation from 10^4^ to 10^5^ BAL cells was performed using NucleoSpin Tissue (Macherey-Nagel) according to the manufacturer’s protocol. To quantify the mtDNA/gDNA ratio, qPCR was used to quantify the mitochondria DNA encoded genes ND1 and ND5 and the haemoglobin B1 gene. Primer sequences are included in Additional file 2: Table S1. Relative amounts of mitochondria DNA to HGB1 genomic sequence was calculated according to 2-^ΔΔCt^ method, where ΔΔCt = (sample Ct GOI-sample Ct HGB1)-(Calibrator Ct GOI- Calibrator Ct HGB1), GOI = gene of interest ie ND1 or ND5, calibrator = mean of all Cts.

### Western blot analysis

1–1.5 million cells were centrifuged and cell pellets were homogenised in RIPA buffer (Invitrogen) containing protease and phosphatase inhibitors, Pierce), followed by storage at -80^ο^C. 40–60 μg Total protein lysates of BAL samples were separated in 12% SDS-PAGE, transferred to 0.45 nm nitrocellulose membrane (Biorad), followed by detection of PINK1[anti-PINK1, mouse monoclonal antibody, (Novus Biologicals)], TOMM20 [anti-TOMM20, rabbit polyclonal (Abnova)], p62 [anti-p62 mouse monoclonal antibody (MBL)], LC3 [anti-LC3 mouse monoclonal antibody (Abgent)] and b-actin [anti-b-actin mouse monoclonal antibody (Sigma)]. Appropriate anti-mouse HRP conjugated secondary antibody (Chemicon) was used and immunodetection was performed with enhanced chemiluminescence reagent Luminata™ (Millipore). Bands were visualised with the ChemiDocXRS+ system (Biorad) and densitometry analyses were performed using Image Lab™ software (Biorad).

### Immunofluorescence

Permeabilisation buffer (0.5%FBS, 0.2%Triton in TBS) was added for 10 min followed by blocking buffer (0.5%FBS, 0.1%Triton, 2 mg/ml BSA in TBS) for 10 min at room temperature. Primary antibody incubations were carried out for 60 min at room temperature followed by washing with TBS. Secondary antibody incubations were performed for 30 min followed by washing with TBS. ToPro or DAPI was added for nuclear staining. Finally, TrueBlack (Biotium) was added for 30 s so as to eliminate autofluorescence signal due to intracellular lipofuscin granules. Images were taken by Leica confocal microscope. Mean intensity of protein levels/cell was performed with ImageJ v2.0.0-rc-69/1.52i. Colocalization of mitochondria with autophagosomes or lysosomes was analysed by coloc2 plugin of Image J software.

### Lysosome inhibition for autophagy and mitophagy flux measurements

BAL cells were plated on glass slides or plastic dishes for 1 h as described above. Following removal of non-adherent cells, AMs were treated with 0.1 mM chloroquine, for 30 min and fixed with 4% formaldehyde for 20 min followed by washing with PBS for immunofluorescence analysis or lysed in RIPA buffer for western blot analyses.

### Statistical analysis

Data were analyzed using Prism 5 (Graph Pad) software and comparisons were made by *t* test, Mann-Whitney test, or chi-square test, as appropriate. Spearman’s correlation coefficient (*r*) analysis measured the association between two variables. A *p* value less than 0.05 was considered statistically significant (*p < 0.05, **p < 0.01, ***p < 0.001).

## Results

### PINK1 and PARK2 expression is reduced in IPF BAL cells

**Fig. 1:**
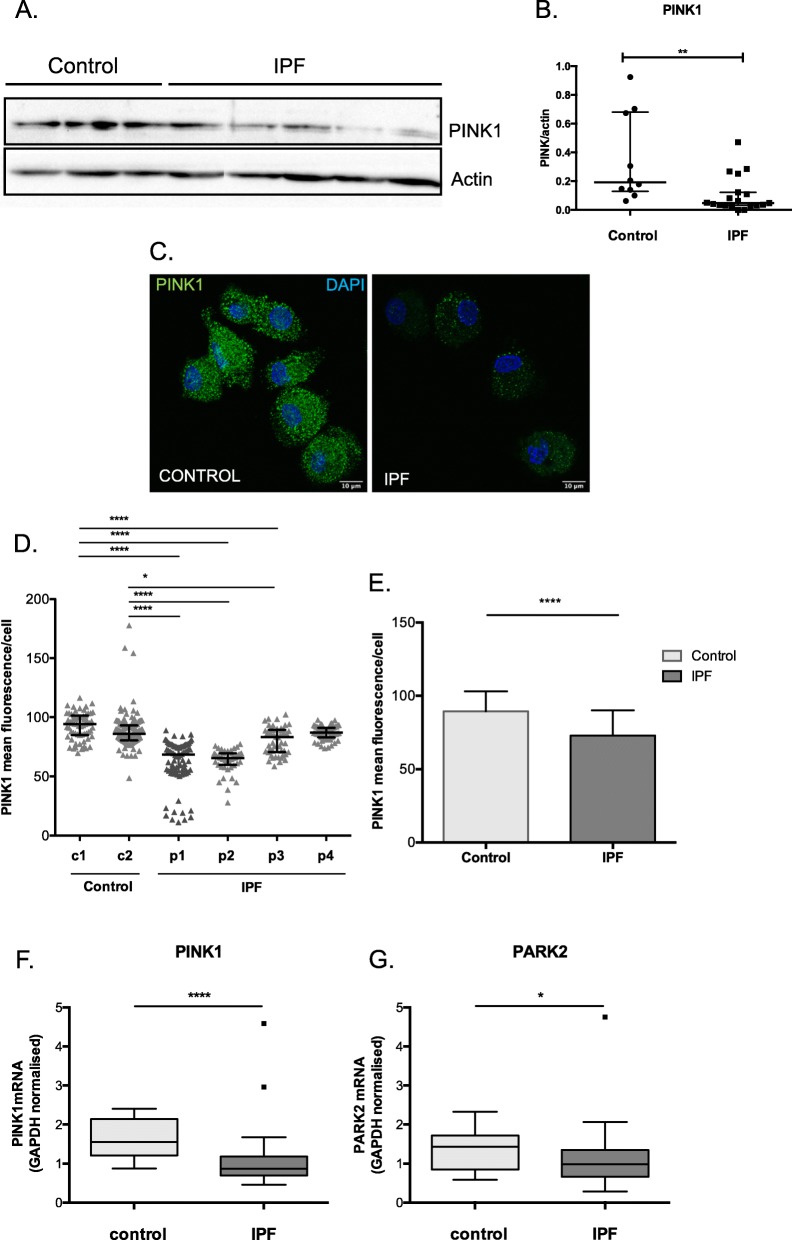
PINK1 expression is reduced in IPF AMs. **a** Representative Western blot and **b** protein levels of PINK1 relative to actin in total BAL cell lysates from IPF (*n* = 19) and control subjects (*n* = 12) (patients characteristics Additional file 2: Table S2). **c** Immune staining of PINK1 in alveolar macrophages/monocytes and PINK1 mean fluorescence/cellper patient (**d)** and group (**e)**. **f** PINK1 and **g** Parkin relative mRNA expression in BALF cells from IPF (*n* = 42) and control (*n* = 14) subjects normalized to GAPDH (patients characteristics Additional file 2: Table S3). (Data represented as median with interquartile range, *p < 0.05, **p < 0.01, Mann-Whitney test)

The downregulation of both PINK1 and PARK2, two key molecules in mitochondrial homeostasis has been reported in IPF [10, 13]. Analysis of total PINK1protein levels in whole BAL cells showed a significant decrease of PINK1 in IPF (Fig. 1a and b). Fluorescence intensity quantification of immune-stained BAL AM cultures also showed lower levels of PINK1 in IPF (Fig. 1c-e). Consistently with protein levels, PINK1 mRNA in total BAL cell lysates were significantly lower in IPF (Fig. 1f). Additionally, PARK2 mRNA was also significantly lower in IPF (Fig. 1g) and positively correlated with PINK1 mRNA levels (*p* < 0.001, *r* = 0.5).

### Increased levels of mtROS in IPF alveolar macrophages

**Fig. 2:**
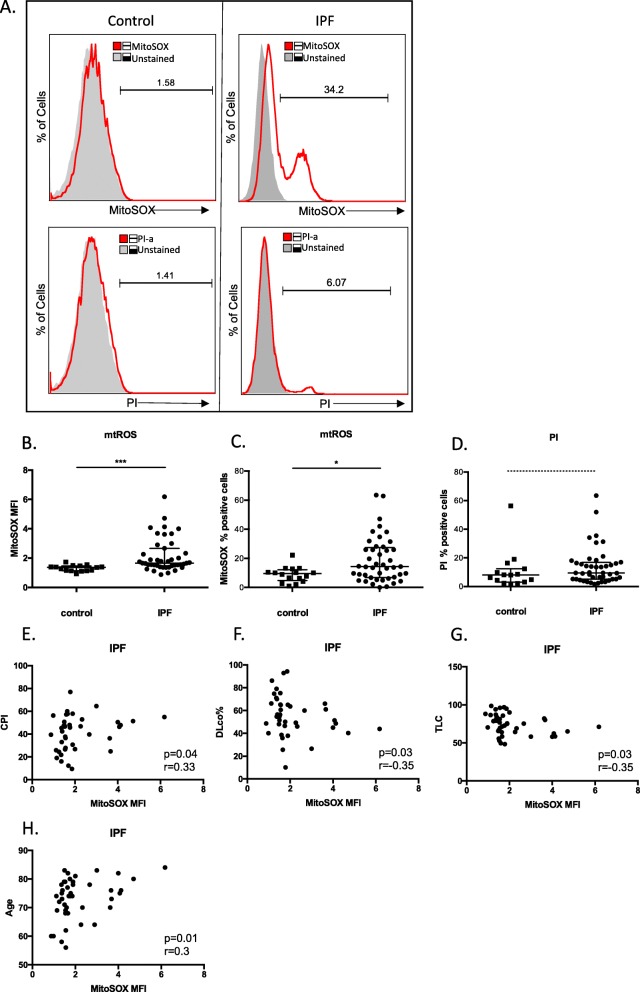
mtROS increases in IPF AMs. **a** Representative histograms of freshly isolated BALF cells from normal and IPF subjects labelled with CD45-FITC and either MitoSOX Red or Propidium Iodide were analysed by flowcytometry. Alveolar macrophages/monocytes were selected (see Additional file 1: Figure S1d for population analysis) and percentage of MitoSOX positive cells and mean fluorescence intensities (red outlined histograms) were analysed relative to CD45-FITC labelled populations (grey histograms). **b** and **c** Median and interquartile range of mean fluorescence intensities (MFI) and percentages of MitoSOX RED positive macrophages from Control (*n* = 15) and IPF (*n* = 43) patients (patients characteristics Additional file 2: Table S4). **d** Median and interquartile range of percentages of Propidium Iodide positive macrophages of matched IPF and normal samples. **e-h** Correlations of mean fluorescence intensities (MFI) of MitoSOX RED positive macrophages from IPF patients with CPI, DLco%, TLC% and age. (Data represented as median with interquartile range*p < 0.05,*** = *p* < 0.001, Mann-Whitney test, *r* = Spearman’scorrelation coefficient)

The levels of mtROS were measured in fresh, BAL AMs (CD45^+^FS^high^SS^high^cells) by flow cytometry using MitoSOX™red superoxide indicator (Fig. 2a). The CD45^+^FS^high^SS^high^ population consisted of CD11c^+^ and/or CD14^+^ macrophages as determined in parallel (Additional file 1: Figure S1c). An increase in mtROS levels was demonstrated in IPF AMs, as shown by higher mean fluorescence intensity (MFI) (Fig. 2b, Additional file 1: Figure S1d) and percentage of positive cells (Fig. 2c). In parallel, analysis of cell viability, using propidium iodide staining (Fig. 2d), revealed no difference between IPF and control groups suggesting that the elevated mtROS in IPF were not due to an increase of apoptotic/necrotic cells.

Importantly, mtROS at the time of diagnosis showed a mild correlation with Composite Physiologic Index (CPI) (*p* = 0.04, spearman *r* = 0.33) (Fig. 2e), lower diffusion capacity for carbon monoxide (DLco%) (*p* = 0.03, spearman *r* = − 0.35)(Fig. 2f) and total lung capacity (TLC%) (*p* = 0.03, spearman *r* = − 0.35)(Fig. 2g). Furthermore, the levels of mtROS significantly correlated with patients’ age (*r* = 0.37, *p* = 0.01) (Fig. 2h) and not smoking habits.

### CD163 expression is upregulated IPF AMs

**Fig. 3:**
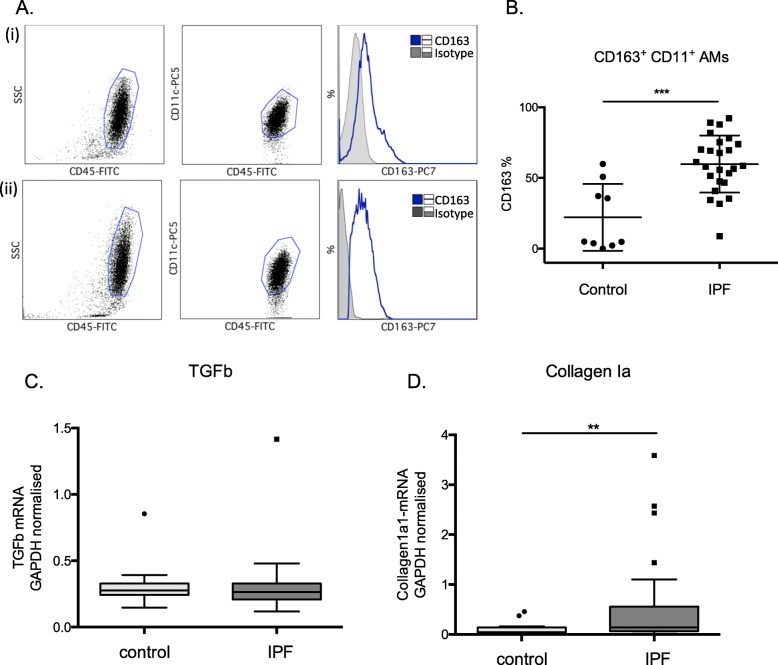
CD163 expression is upregulated IPF AMs. **a** Representative examples of (i) control, and (ii) IPF BAL samples stained with CD45/CD11c/CD163. CD163 levels were measured in the CD45^+^FS^high^SS^high^CD11c. **b** Percentages of CD163^+^CD11^+^ cells in BAL samples from controls (*n* = 9) and IPF (*n* = 25) (patients characteristics Additional file 2: Table S5). **c** TGF and **d** Collagen1a1 relative mRNA expression in BALF cells from IPF (*n* = 42) and control (*n* = 14) subjects normalized to GAPDH (patients characteristics Additional file 2: Table S3). (Data represented as median with interquartile range *** = *p* < 0.001, Mann-Whitney test)

MtROS is required for alternative activation of macrophages. We therefore examined the surface expression of scavenger receptor CD163, a frequently used marker of alternatively activated macrophages [25] by flow cytometry in the CD45^+^FS^high^SS^high^CD11c^+^AM population of the BAL cells. CD163 expression was significantly higher in IPF relative to controls (Fig. 3a and b). However, no correlation between CD163 and the levels of mtROS in the IPF cohort (*r* mtROS-MFI = -0.11, *r* mtROS-% = − 0.27, *p*NS) was observed. TGFb mRNA expression was not higher in IPF BAL cells (Fig. 3c), in contrast to Collagen1a levels (Fig. 3d) as previously described [24].

### Mitochondria encoded OXPHOS related gene expression is lower in IPF BAL cells

**Fig. 4:**
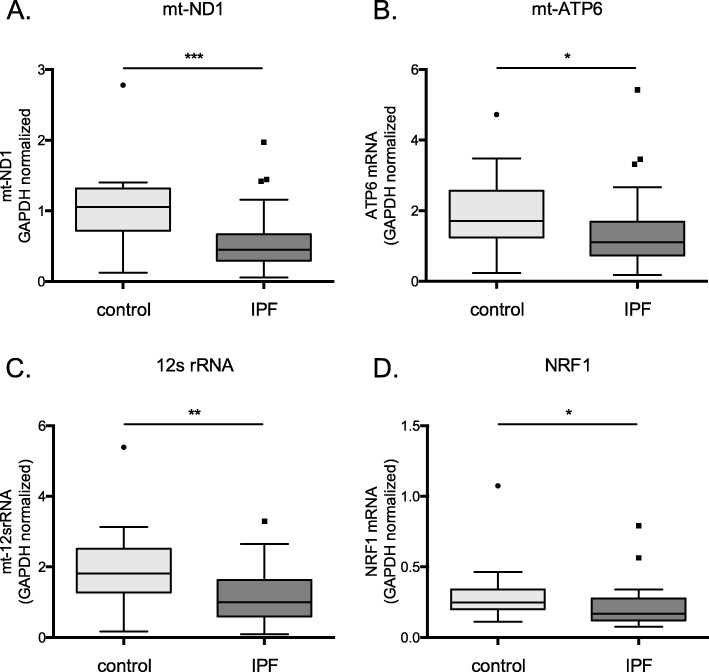
Mitochondria encoded OXPHOS expression is lower in IPF BAL cells. **a** mt-ND1 **b** mt-ATP6, **c** 12srRNA and **d** NRF1 relative mRNA expression in BALF cells from IPF (*n* = 42) and control (*n* = 14) subjects normalized to GAPDH (patients characteristics Additional file 2: Table S4). (Data represented as median with interquartile range, *p < 0.05, **p < 0.01, Mann-Whitney test)

Persistently elevated levels of mtROS observed during ageing and chronic lung diseases such as IPF may drive mitochondria damage and impair their function. Since alternatively activated macrophages rely on OXPHOS for ATP production, we analyzed the transcription of mt-encoded gene members of OXPHOS in particular. Transcription of MRC complex I and V members, NADH dehydrogenase subunit 1 (ND1) and ATP synthase 6 (ATP6) respectively, was significantly lower in IPF BAL cells (Fig. 4a and b). Furthermore, the mt-encoded 12 s ribosomal RNA required for the intra-mitochondrial translation was also significantly decreased in IPF (Fig. 4c). Additionally, the expression of nuclear respiratory factor 1(NRF1), that activates the expression of mitochondrial transcription factor A (TFAM) and in turn mt-OXPHOS gene expression, was also significantly lower in IPF BAL cells (Fig. 4d). Importantly, the levels of mtROS were associated with a reduction of MRC transcription of both complex I (ND1*r* = − 0.37, *p* = 0.03) and complex V (ATP6 *r* = − 0.4,*p* = 0.01).

### Accumulation of dysmorphic mitochondria in IPF AMs

**Fig. 5:**
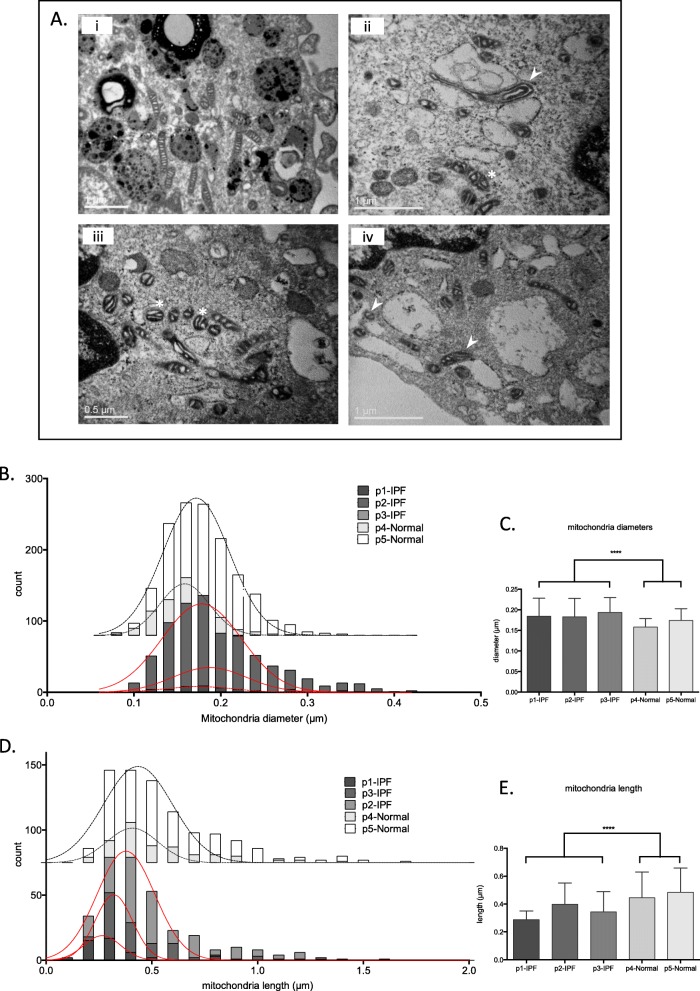
Dysmorphic mitochondria in IPF AMs **a** Representative TEM images (i: control, ii/iii/iv: IPF) of alveolar macrophages showing swollen and dysmorphic mitochondria in IPF patients (*) as well as vesicle associated mitochondria (➤) Scale bars = 1 μm. Frequency and medians of mitochondria diameters (**b**-**c**) and mitochondria length (**d-e**) in control (*n* = 2) and IPF (*n* = 3) macrophages

Mitochondria morphology in IPF AMs was evaluated using transmission electron microscopy (TEM). Ultrastructural qualitative analysis revealed that mitochondria in IPF were dysmorphic and contained disorganized cristae (Fig. 5a). Quantitative morphometry in a subset of samples showed a significant increase in the frequency of swollen mitochondria with higher median diameter size ascompared to controls (Fig. 5b and c). In parallel, in IPF, mitochondria were shorteras shown by their significantly decreased length (Fig. 5d and e).

### Mitochondria mass was not altered in IPF AMs

Previous studies suggested that mitochondria mass increases in IPF lungs [10, 26]. We therefore determined the levels of mtDNA and mitochondria protein marker, Translocase Of Outer Mitochondrial Membrane 20 (TOMM20). The mitochondria encoded genes, ND1 and ND5, relative to the hemoglobin 1 (HGB1) genomic locus were analyzed by qPCR in whole BAL cell lysates. No differences between controls and IPF patients (Additional file 3: Figure S2a and b), or mtDNA correlation with mtROS were observed. Additionally, TOMM20 levels, measured either by western blot in whole BAL cell lysates or immune fluorescence in AMs, were similar between the two groups (Additional file 3: Figure S2c-g).

### LC3B II and autophagy levels in IPF alveolar macrophages

**Fig. 6:**
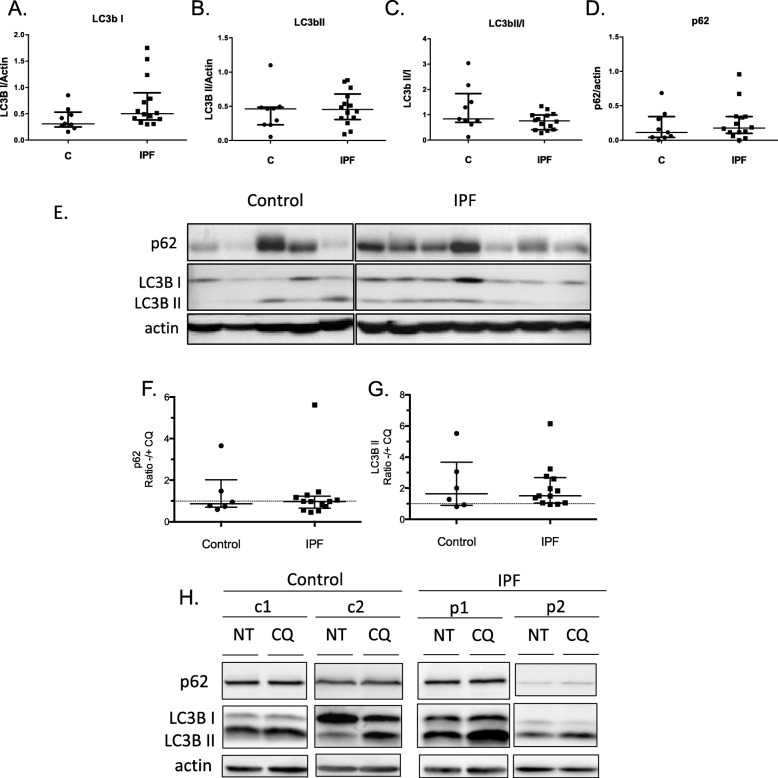
Autophagy markers in IPF AMs. Protein levels of LC3B I (**a**), LC3B II (**b**) LC3BII/I (**c**), p62 (**d**) relative to actin in total BAL cell lysates from IPF (*n* = 16) and control (*n* = 9) subjects. **e** Corresponding Western blot. Protein levels of p62 (**f**) and LC3B II (**g**) in BAL cells treated with Chloroquine (CQ) for 1 h relative to untreated (NT) from IPF (*n* = 13) and controls (*n* = 6). **h** Representative Western Blots of control and IPF samples. For all comparisons Mann-Whitney test was used, data are represented as median with interquartile ranges

In view of the accumulation of oxidized and dysmorphic mitochondria in AMs we subsequently examined commonly used markers of autophagy and mitophagy. Initially, we assessed endogenous basal expression of autophagy markers; microtubule-associated protein light chain 3B (LC3B), forms I and II, and the selective autophagy receptor p62/sequestosome1 (SQSTM1) protein in BAL cells. Western Blot analysis from IPF and control groups showed no significant differences in the cytosolic form of LC3B (LC3B I), the lipidated form present mostly on autophagosomes (LC3B II), the LC3B II/I ratio or the p62 protein (Fig. 6a-e and Additional file 4: Figure S3a-b). mRNA levels of p62 and Beclin 1 were also measured and no significant differences were observed (Additional file 4: Figure S3c-d).

Autophagy flux was measured following treatment with chloroquine, a lysosomal acidification inhibitor that enables the estimation of the turnover of proteins participating in the degradation of autophagy cargo. Similarly to the basal levels, we did not observe significant differences in the accumulation of autophagy markers LC3B II and p62 in IPF relative to controls (Fig. 6f-h). LC3B levels were also analysed in AM cultures, following chloroquine treatment. In agreement with the western blot analysis, in addition to LC3B I, LC3B II puncta were readily observed in control and IPF AMs, in untreated and following chloroquine treatment (Additional file 4: Figure S3e).

### LC3B/PINK1 dependent mitophagy levels were similar in IPF and controls

**Fig. 7:**
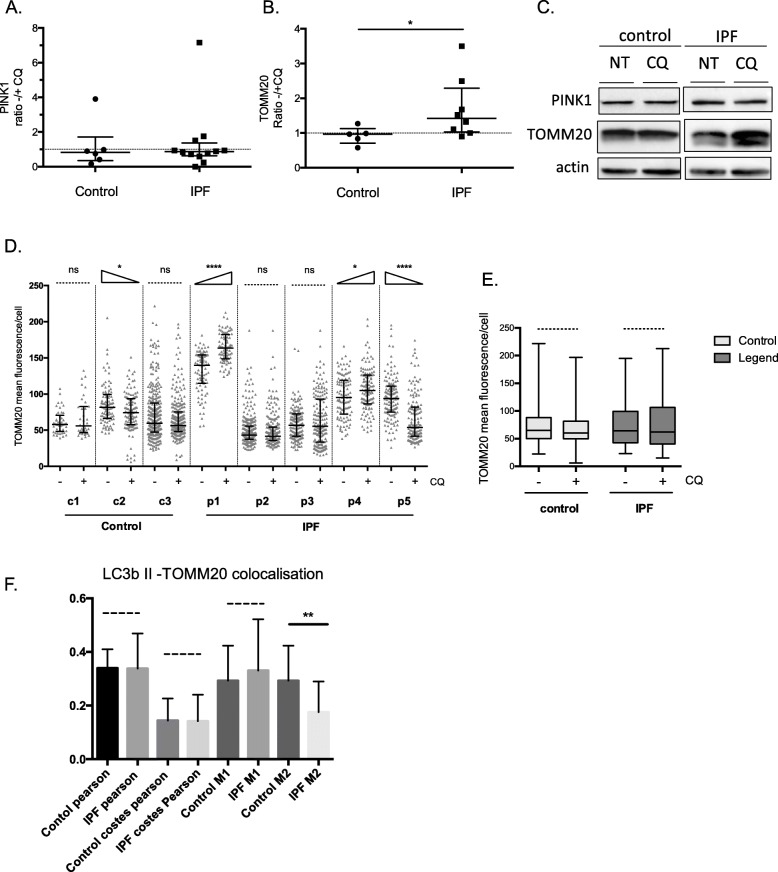
LC3B/PINK1 dependent mitophagy levels in IPF AMs. Protein levels of PINK1 (**a**) and TOMM20 (**b**) in BAL cells treated with Chloroquine (CQ) for 1 h relative to untreated (NT) from IPF (*n* = 12 and *n* = 8 respectively) and control (*n* = 6 and *n* = 5 respectively). **c** Representative Western Blots of control and IPF samples. **d** and **e** TOMM20 mean fluorescence intensity/cell measured from images acquired by confocal microscopy, per patient and group respectively. **f** TOMM20-LC3B II Pearson coefficient of colocalization, Costes’s threshold corrected Pearson coefficient of colocalization and M1 and M2 Menders colocalization values comparisons of IPF and control groups following CQ treatment. For all comparisons Mann-Whitney test was used except for colocalizations where t-test was used. Data are represented as median with interquartile ranges. See methods for Western blot and immune fluorescence analyses

As an indication of mitophagy induction and mitochondria degradation rate, PINK1 and TOMM20 turnover was measured following lysosome inhibition. PINK1 levels did not show any increase following chloroquine treatment (Fig. 7a and c) while TOMM20 accumulation varied among IPF patients and overall increased significantly (Fig. 7b and c). In AMs significant variation in TOMM20 accumulation following lysosome inhibition was observed while, overall no significant increase was observed among the two groups (Fig. 7d-e).

Additionally, the colocalization of TOMM20 with LC3B II puncta in AMs, by immunofluorescence and confocal microscopy was analyzed, as a more direct assessment of mitophagy. TOMM20 and LC3B II colocalization was not more prominent in IPF than controls following chloroquine treatment. This was confirmed by the lack of significant differences in the colocalization coefficients’ analysis that included Pearson coefficient of colocalization, Costes’s threshold corrected Pearson coefficient of colocalization and M1 and M2 Menders colocalization values(Fig. 7f).

## Discussion

Impaired mitochondria homeostasis and function are established hallmarks of aging and contribute to cell senescence, chronic inflammation and age-dependent decline in stem cell activity [27] while age associated changes have been linked to IPF pathogenesis [3, 28]. In this study, we used BAL, a tool for both clinical and research purposes [29, 30], and a rich source of AMs. We showed that mitochondria in AMs from IPF patients have prominent morphological defects and impaired transcription. We observed a significant reduction of mitochondria homeostasis regulators PINK1, PARK2 and NRF1. Additionally, a significant increase in mtROS was associated with reduced expression of mitochondria-encoded OXPHOS genes.

PINK1 plays a significant role in mitochondria homeostasis via mitophagy dependent and independent pathways [31]. A decrease in PINK1 levels in type II AECs from IPF patients, IPF lung tissue, aging mice and mice challenged with bleomycin has been associated with the development of fibrosis [9–12, 32]. PINK1-knockout results in increased mtROS production, mitochondria fragmentation/fission and activation of autophagy, prompting the clearance of oxidized mitochondria [9, 33]. We demonstrated a significant decrease in PINK1 levels, coupled to lower PARK2 transcription, in IPF AMs. Consistently with decreased PINK1 and increased mtROS, morphological characteristics of mitochondria, including prominent association with vesicular structures, swollen, irregular shapes, shorter length, and disorganized cristae, suggestive of mitochondrial fission, were also observed.

The accumulation of oxidized mitochondria could be attributed to a dysregulation of mitochondria quality control mechanism that includes clearance of damaged mitochondria mainly via mitophagy [34]. Autophagosome formation and LC3B activation were previously shown reduced in IPF lungs and following TGF-βtreatment [35], while fibroblast foci show elevated activation of mTOR, suggesting a downregulation of autophagy [36]. However, a survival mechanism of pro-fibrotic AMs via increased LC3B-dependent mitophagy of PINK1 associated mitochondria has been previously demonstrated [37]. In our study, the endogenous levels of LC3B II were quite prominent in several samples suggesting the presence of the lipid-associated form of LC3B in AMs from both controls and IPF. We did not observe increased levels of LC3B II in IPF as previously shown [37], while autophagy flux was similar in the two groups. Furthermore, we assessed mitophagy levels by the degree of colocalization of LC3B puncta with mitochondrial protein TOMM20 and no significant differences were observed. Interestingly, we observed a significant increase in lysosomal degradation of TOMM20 in IPF cells. Conceivably, the increased levels of mitochondria degradation could be attributed to increased phagocytosis of cell free mitochondria accumulated in BAL [26]. Alternatively, our results may reflect an imbalance in mitochondria biogenesis and degradation, since a recent study has shown that the biogenesis of mitochondria is also defective in IPF [11]. Additionally, other mitochondria clearance mechanisms have been described such as mitochondria derived vesicles and spheroids that drive mitochondria lysosomal degradation independent of the classic autophagy pathway and may play a role in IPF [38].

Furthermore, mtROS was significantly increased and associated with increased age and diminished lung function in IPF, suggesting a correlation with disease severity. Persistently increased mtROS, has been associated with mitochondria damage that could blunt macrophage activation and function. Indeed, increased basal levels of mtROS in Chronic Obstructive Pulmonary Disease (COPD) resulted in lower acute mtROS production upon bacterial infection leading to reduced apoptosis and bactericidal activity of AMs [39]. Notably, the persistently increased mtROS in IPF AMs described in this study may explain previous observations showing that innate immune responses such as NLRP3 inflammasome activation and antiviral gene expression are altered in the BAL cells of IPF patients [40, 41].

Immune cell functions including macrophage plasticity are largely shaped by mitochondria activity and cellular metabolism [4, 42]. Activation of Toll-like receptors, such as TLR4, leads to a switch from OXPHOS to glycolysis in immune cells, similar to that occurring in tumors [43] whereas macrophages activated with IL-4 commit to OXPHOS and fatty acid oxidation (FAO) showing minimal metabolic adaptation when compared to homeostasis [42]. Two populations of AMs exist in the injured lung, resident AMs, and recruited AMs that originate from circulating monocytes. Recruited AMs produce inflammatory cytokines in association with increased glycolytic and arginine metabolism whereas resident AMs proliferate locally and are characterized by increased tricarboxylic acid cycle and amino acid metabolism [44]. Pro-fibrotic mouse AMs showed an increase in glycolysis and glycolytic enzyme transcription, but no dependence on FAO or OXPHOS [45]. Importantly, reversal of macrophage phenotype by 2-deoxy-D-glucose, an inhibitor of glycolysis was shown to reduce fibrosis in the same study [45]. To date, there are no reports of macrophage metabolic profiles from IPF patients. Interestingly, expression of MRC I and V mt-encoded OXPHOS genes was significantly reduced in IPF BAL cells. In addition, the NRF1 transcription factor that regulates mt-respiratory gene expression and mitochondria biogenesis [46] was significantly downregulated in IPF. Intriguingly, loss of PINK1 also results in decreased MRC complex 1 activity independently of mitophagy [47]. Therefore, our results may suggest a decrease in mitochondria respiratory activity in IPF alveolar macrophages.

According to the previous paradigm of macrophage categorisation into classical/proinflammatory, alternative/resolving and pro-repair/fibrotic population, alternatively activated macrophages were suggested to predominate in IPF as indicated by the increased surface expression of manose receptor CD206 [48]. In our study, the levels of CD45^+^CD11c^+^CD163^+^ alveolar macrophages were significantly higher in IPF BAL. Recent studies propose that a diverse population of macrophages exists in IPF lungs with both pro-inflammatory and pro-fibrotic gene expression [18, 19, 49, 50]. Further studies are required to elucidate the phenotypic and functional characteristics of the AM population in the BAL showing increased mtROS.

Some limitations of our study should be considered, mainly regarding the differences in the number of patients included in each experiment, which is based on the number of cells required for each method, and the diversity in total cell count of cells in each BAL sample. However, it needs to be stressed that patients were randomly selected for each method, and furthermore, mtROS levels were evaluated in most patients included in the study.

The impact of dysregulated mitochondrial complex expression and increased oxidation levels on the antimicrobial action of lung macrophages in health and disease has been recently described [51–53]. Furthermore, the association of increased mtROS with aging, a major cofactor in the development of IPF was evident in this series of patients. Our study provides novel findings about mitochondrial gene expression, morphology, and oxidation levels, in alveolar macrophages in IPF that could be indirectly linked with IPF pathogenesis and disease progression. Conclusively, we report that AMs in IPF accumulate oxidized and dysmorphic mitochondria, which correlated with clinical variables of the disease. Impaired mitochondria activity may provide a better understanding of AMs activation in IPF. Importantly, targeted antioxidant therapies may need re-evaluation in IPF with respect to macrophage functions.

## Supplementary information

**Additional file 1: Figure S1a.** A549 cells treated with 1000 μM H2O2 were stained with 5 μm MitoSOXTMred and examined using confocal microscopy. The characteristic mitochondrial network/web staining in the cytoplasm observed, was significantly enhanced upon H202 treatment. **Figure S1b.** PMA treated THP1 cells were stained with 5 μm MitoSOXTMred or Propidium Iodide and analysed by flow cytometry. The non-treated PI negative cell population stained positive with MitoSOX while treatment with 1000 μM H2O2 resulted in stronger MitoSOX staining (pale pink histograms). Following H2O2 treatment the PI positive population showed the highest MitoSOX staining (dark red histogram). **Figure S1c**. Representative example of BAL sample stained with CD45-FITC/CD11c-PC5 or CD45-FITC/CD14-PC. The CD45 + FShighSShigh population in panel (i) comprises of CD11c positive cells (panel ii) and a lower percentage of CD14 positive cells (iii). The CD45 + FShighSShigh population of alveolar macrophages/monocyte derived phagocytes, was used for MitoSOXTMred analyses. **Figure S1d.** Representative examples of control and IPF BAL MitoSOX analyses.

**Additional file 2: Table S1.** Primer sequences for qPCR. **Table S2.** Clinicopathological characteristics of the subjects included in Fig. 1b. Values are expressed as means±SD or medians with range. **Table S3.** Clinicopathological characteristics of the subjects included in Fig. 1f and g, Fig. 3c-d and Fig. 4a-d. Values are expressed as means±SD or medians with range. **Table S4.** Clinicopathological characteristics of the subjects included in Fig. 2b-d Values are expressed as means±SD or medians with range. **Table S5.** Clinicopathological characteristics of the subjects included in Fig. 3b. Values are expressed as means±SD or medians with range.

**Additional file 3: Figure S2.** Mitochondrial DNA content assessed by mtDNA/gDNA ratio measuring both ND1(A) and ND5(B) relative to HGB1 in BAL cells. No statistical difference between control (*n* = 10) and IPF (*n* = 18). Representative western blot (C) and densitometry analysis (D) of mitochondria protein TOMM20 showing no statistical difference between control (*n* = 5) and IPF (*n* = 11). TOMM20 mean fluorescence intensity/cell measured from images acquired by confocal microscopy, per patient (E) and group (F). Non significant differences are highlighted by doted lines, all other comparisons showed statistically significant differences according to Kruskal Wallis test with Dunn’s test for multiple comparisons. (G) Representative image of TOMM20 staining of alveolar macrophages from IPF patient (p1). With the exception of (E) all for all other comparisons Mann-Whitney test was used, data are represented as median with interquartile ranges.

**Additional file 4: Figure S3.** A, B: (i) LC3b I, (ii)II and (iii) LC3bII/I densitometry analyses from western blots performed on 2 independent IPF/control cohorts, C and D: relative mRNA expression of p62 and Beclin1. E. Representative images of LC3b immunostained alveolar macrophages from IPF and control samples treated with CQ or left untreated. All graphs represent median and interquartile ranges, no significant differences observed according to Mann-Whitney test.

## Data Availability

The datasets used and/or analysed during the current study are available from the corresponding author on reasonable request.

## Abbreviations

AMs: Alveolar macrophages
BAL: Bronchoalveolar lavage
BECN1: Beclin-1coiled-coil myosin-like BCL2-interacting protein
GAPDH: Glyceraldehyde-3-phosphate dehydrogenase
HGB1: Haemoglobin 1 gene
IPF: Idiopathic pulmonary fibrosis
MRC: Mitochondria respiratory chain
MRC: Mitochondria respiratory chain
MT: Mitochondrial
MT-ATP6: ATP synthase 6
MT-ND1: Mitochondrial encoded NADH dehydrogenase 1
MT-ND5: Mitochondrial encoded NADH dehydrogenase 5
NRF1: Nuclear respiratory factor 1
OXPHOS: Oxidative phosphorylation
p62: Selective autophagy receptor p62/sequestosome1 SQSTM1
PARK2: Parkin E3 ubiquitin ligase
PINK1: PTEN-induced kinase 1
ROS: Reactive oxygen species
rRNA: ribosomal RNA

## References

[1] Richeldi L, Collard HR, Jones MG (2017). Idiopathic pulmonary fibrosis. Lancet.

[2] Wuyts WA, Agostini C, Antoniou KM, Bouros D, Chambers RC, Cottin V (2013). The pathogenesis of pulmonary fibrosis: a moving target. Eur Respir J.

[3] Zank DC, Bueno M, Mora AL, Rojas M (2018). Idiopathic pulmonary fibrosis: aging, mitochondrial dysfunction, and cellular bioenergetics. Front Med.

[4] Angajala A, Lim S, Phillips JB, Kim J-H, Yates C, You Z (2018). Diverse Roles of Mitochondria in Immune Responses: Novel Insights Into Immuno-Metabolism. Front Immunol.

[5] Bratic A, Larsson N-G (2013). The role of mitochondria in aging. J Clin Invest.

[6] Palikaras K, Lionaki E, Tavernarakis N (2015). Balancing mitochondrial biogenesis and mitophagy to maintain energy metabolism homeostasis. Cell Death Differ.

[7] Palikaras K, Tavernarakis N (2014). Mitochondrial homeostasis: the interplay between mitophagy and mitochondrial biogenesis. Exp Gerontol.

[8] Mora AL, Bueno M, Rojas M (2017). Mitochondria in the spotlight of aging and idiopathic pulmonary fibrosis. J Clin Invest.

[9] Patel AS, Song JW, Chu SG, Mizumura K, Osorio JC, Shi Y (2015). Epithelial cell mitochondrial dysfunction and PINK1 are induced by transforming growth factor-beta1 in pulmonary fibrosis. PLoS One.

[10] Bueno M, Lai YC, Romero Y, Brands J, St Croix CM, Kamga C (2015). PINK1 deficiency impairs mitochondrial homeostasis and promotes lung fibrosis. J Clin Invest.

[11] Yu G, Tzouvelekis A, Wang R, Herazo-Maya JD, Ibarra GH, Srivastava A (2018). Thyroid hormone inhibits lung fibrosis in mice by improving epithelial mitochondrial function. Nat Med.

[12] Sosulski ML, Gongora R, Danchuk S, Dong C, Luo F, Sanchez CG (2015). Deregulation of selective autophagy during aging and pulmonary fibrosis: the role of TGFbeta1. Aging Cell.

[13] Kobayashi K, Araya J, Minagawa S, Hara H, Saito N, Kadota T (2016). Involvement of PARK2-Mediated Mitophagy in Idiopathic Pulmonary Fibrosis Pathogenesis. J Immunol.

[14] Daniil Z, Kotsiou OS, Grammatikopoulos A, Peletidou S, Gkika H, Malli F (2018). Detection of mitochondrial transfer RNA (mt-tRNA) gene mutations in patients with idiopathic pulmonary fibrosis and sarcoidosis. Mitochondrion..

[15] Sato N, Takasaka N, Yoshida M, Tsubouchi K, Minagawa S, Araya J (2016). Metformin attenuates lung fibrosis development via NOX4 suppression. Respir Res.

[16] Murray LA, Chen Q, Kramer MS, Hesson DP, Argentieri RL, Peng X (2011). TGF-beta driven lung fibrosis is macrophage dependent and blocked by serum amyloid P. Int J Biochem Cell Biol.

[17] Zhou X, Moore BB. Location or origin? What is critical for macrophage propagation of lung fibrosis? Eur Respir J. 2018;51(3). 10.1183/13993003.00103-2018 PMC6383715 29496789

[18] Aran D, Looney AP, Liu L, Wu E, Fong V, Hsu A (2019). Reference-based analysis of lung single-cell sequencing reveals a transitional profibrotic macrophage. Nat Immunol.

[19] Misharin AV, Morales-Nebreda L, Reyfman PA, Cuda CM, Walter JM, McQuattie-Pimentel AC (2017). Monocyte-derived alveolar macrophages drive lung fibrosis and persist in the lung over the life span. J Exp Med.

[20] Zhang Y, Choksi S, Chen K, Pobezinskaya Y, Linnoila I, Liu ZG (2013). ROS play a critical role in the differentiation of alternatively activated macrophages and the occurrence of tumor-associated macrophages. Cell Res.

[21] Osborn-Heaford HL, Ryan AJ, Murthy S, Racila A-M, He C, Sieren JC (2012). Mitochondrial Rac1 GTPase import and Electron transfer from cytochrome c are required for pulmonary fibrosis. J Biol Chem.

[22] Raghu G, Collard HR, Egan JJ, Martinez FJ, Behr J, Brown KK (2011). An official ATS/ERS/JRS/ALAT statement: idiopathic pulmonary fibrosis: evidence-based guidelines for diagnosis and management. Am J Respir Crit Care Med.

[23] Samara KD, Trachalaki A, Tsitoura E, Koutsopoulos AV, Lagoudaki ED, Lasithiotaki I (2017). Upregulation of citrullination pathway: from autoimmune to idiopathic lung fibrosis. Respir Res.

[24] Tsitoura E, Wells AU, Karagiannis K, Lasithiotaki I, Vasarmidi E, Bibaki E (2016). MiR-185/AKT and miR-29a/collagen 1a pathways are activated in IPF BAL cells. Oncotarget..

[25] Kaku Y, Imaoka H, Morimatsu Y, Komohara Y, Ohnishi K, Oda H (2014). Overexpression of CD163, CD204 and CD206 on alveolar macrophages in the lungs of patients with severe chronic obstructive pulmonary disease. PLoS One.

[26] Ryu C, Sun H, Gulati M, Herazo-Maya JD, Chen Y, Osafo-Addo A (2017). Extracellular mitochondrial DNA is generated by fibroblasts and predicts death in idiopathic pulmonary fibrosis. Am J Respir Crit Care Med.

[27] Sun N, Youle RJ, Finkel T (2016). The mitochondrial basis of aging. Mol Cell.

[28] Antoniou KM, Hansell DM, Rubens MB, Marten K, Desai SR, Siafakas NM (2008). Idiopathic pulmonary fibrosis: outcome in relation to smoking status. Am J Respir Crit Care Med.

[29] Raghu G, Remy-Jardin M, Myers JL, Richeldi L, Ryerson CJ, Lederer DJ (2018). Diagnosis of idiopathic pulmonary fibrosis. An official ATS/ERS/JRS/ALAT clinical practice guideline. Am J Respir Crit Care Med.

[30] Prasse A, Binder H, Schupp JC, Kayser G, Bargagli E, Jaeger B (2019). BAL cell gene expression is indicative of outcome and airway basal cell involvement in idiopathic pulmonary fibrosis. Am J Respir Crit Care Med.

[31] Voigt A, Berlemann LA, Winklhofer KF (2016). The mitochondrial kinase PINK1: functions beyond mitophagy. J Neurochem.

[32] Bueno M, Brands J, Voltz L, Fiedler K, Mays B, St Croix C, et al. ATF3 represses PINK1 gene transcription in lung epithelial cells to control mitochondrial homeostasis. Aging Cell. 2018;17(2). 10.1111/acel.12720 PMC5847866 29363258

[33] Dagda RK, Cherra SJ, Kulich SM, Tandon A, Park D, Chu CT (2009). Loss of PINK1 function promotes mitophagy through effects on oxidative stress and mitochondrial fission. J Biol Chem.

[34] Hara H, Kuwano K, Araya J. Mitochondrial quality control in COPD and IPF. Cells. 2018;7(8). 10.3390/cells7080086 PMC6115906 30042371

[35] Patel AS, Lin L, Geyer A, Haspel JA, An CH, Cao J (2012). Autophagy in idiopathic pulmonary fibrosis. PLoS One.

[36] Gui YS, Wang L, Tian X, Li X, Ma A, Zhou W (2015). mTOR Overactivation and compromised autophagy in the pathogenesis of pulmonary fibrosis. PLoS One.

[37] Larson-Casey JL, Deshane JS, Ryan AJ, Thannickal VJ, Carter AB (2016). Macrophage Akt1 kinase-mediated Mitophagy modulates apoptosis resistance and pulmonary fibrosis. Immunity..

[38] McLelland GL, Soubannier V, Chen CX, McBride HM, Fon EA (2014). Parkin and PINK1 function in a vesicular trafficking pathway regulating mitochondrial quality control. EMBO J.

[39] Bewley MA, Preston JA, Mohasin M, Marriott HM, Budd RC, Swales J (2017). Impaired mitochondrial Microbicidal responses in chronic obstructive pulmonary disease macrophages. Am J Respir Crit Care Med.

[40] Lasithiotaki I, Giannarakis I, Tsitoura E, Samara KD, Margaritopoulos GA, Choulaki C (2016). NLRP3 inflammasome expression in idiopathic pulmonary fibrosis and rheumatoid lung. Eur Respir J.

[41] Lasithiotaki I, Antoniou KM, Vlahava VM, Karagiannis K, Spandidos DA, Siafakas NM (2011). Detection of herpes simplex virus type-1 in patients with fibrotic lung diseases. PLoS One.

[42] O'Neill LA, Pearce EJ (2016). Immunometabolism governs dendritic cell and macrophage function. J Exp Med.

[43] Rodriguez-Prados JC, Traves PG, Cuenca J, Rico D, Aragones J, Martin-Sanz P (2010). Substrate fate in activated macrophages: a comparison between innate, classic, and alternative activation. J Immunol.

[44] Mould KJ, Barthel L, Mohning MP, Thomas SM, McCubbrey AL, Danhorn T (2017). Cell origin dictates programming of resident versus recruited macrophages during acute lung injury. Am J Respir Cell Mol Biol.

[45] Xie N, Cui H, Ge J, Banerjee S, Guo S, Dubey S (2017). Metabolic characterization and RNA profiling reveal glycolytic dependence of profibrotic phenotype of alveolar macrophages in lung fibrosis. Am J Physiol Lung Cell Mol Physiol.

[46] Vercauteren K, Pasko RA, Gleyzer N, Marino VM, Scarpulla RC (2006). PGC-1-related coactivator: immediate early expression and characterization of a CREB/NRF-1 binding domain associated with cytochrome c promoter occupancy and respiratory growth. Mol Cell Biol.

[47] Morais VA, Haddad D, Craessaerts K, De Bock PJ, Swerts J, Vilain S (2014). PINK1 loss-of-function mutations affect mitochondrial complex I activity via NdufA10 ubiquinone uncoupling. Science..

[48] Pechkovsky DV, Prasse A, Kollert F, Engel KM, Dentler J, Luttmann W (2010). Alternatively activated alveolar macrophages in pulmonary fibrosis-mediator production and intracellular signal transduction. Clin Immunol.

[49] Lee J, Arisi I, Puxeddu E, Mramba LK, Amicosante M, Swaisgood CM (2018). Bronchoalveolar lavage (BAL) cells in idiopathic pulmonary fibrosis express a complex pro-inflammatory, pro-repair, angiogenic activation pattern, likely associated with macrophage iron accumulation. PLoS One.

[50] Allden SJ, Ogger PP, Ghai P, McErlean P, Hewitt R, Toshner R (2019). The transferrin receptor CD71 delineates functionally distinct airway macrophage subsets during idiopathic pulmonary fibrosis. Am J Respir Crit Care Med.

[51] Plataki M, Cho SJ, Harris RM, Huang HR, Yun HS, Schiffer KT (2019). Mitochondrial dysfunction in aged macrophages and lung during primary Streptococcus pneumoniae infection is improved with Pirfenidone. Sci Rep.

[52] Liu Y, Lu F, Kang L, Wang Z, Wang Y (2017). Pirfenidone attenuates bleomycin-induced pulmonary fibrosis in mice by regulating Nrf2/Bach1 equilibrium. BMC Pulm Med.

[53] Belchamber KBR, Singh R, Batista CM, Whyte MK, Dockrell DH, Kilty I, et al. Defective bacterial phagocytosis is associated with dysfunctional mitochondria in COPD macrophages. Eur Respir J. 2019;54. 10.1183/13993003.02244-2018 31320451

