# Supplementary material for: Accumulation of damaged mitochondria in alveolar macrophages with reduced OXPHOS related gene expression in IPF
Source: Respir Res. 2019 Nov 27;20:264. doi: 10.1186/s12931-019-1196-6 (PMC6880424; doi:10.1186/s12931-019-1196-6)
Supplement: Supplementary file 1 — Additional file 1: Figure S1a. A549 cells treated with 1000 μM H2O2 were stained with 5 μm MitoSOXTMred and examined using confocal microscopy. The characteristic mitochondrial network/web staining in the cytoplasm observed, was significantly enhanced upon H202 treatment. Figure S1b. PMA treated THP1 cells were stained with 5 μm MitoSOXTMred or Propidium Iodide and analysed by flow cytometry. The non-treated PI negative cell population stained positive with MitoSOX while treatment with 1000 μM H2O2 resulted in stronger MitoSOX staining (pale pink histograms). Following H2O2 treatment the PI positive population showed the highest MitoSOX staining (dark red histogram). Figure S1c. Representative example of BAL sample stained with CD45-FITC/CD11c-PC5 or CD45-FITC/CD14-PC. The CD45 + FShighSShigh population in panel (i) comprises of CD11c positive cells (panel ii) and a lower percentage of CD14 positive cells (iii). The CD45 + FShighSShigh population of alveolar macrophages/monocyte derived phagocytes, was used for MitoSOXTMred analyses. Figure S1d. Representative examples of control and IPF BAL MitoSOX analyses. [file 12931_2019_1196_MOESM1_ESM.pptx]

## Slide 1
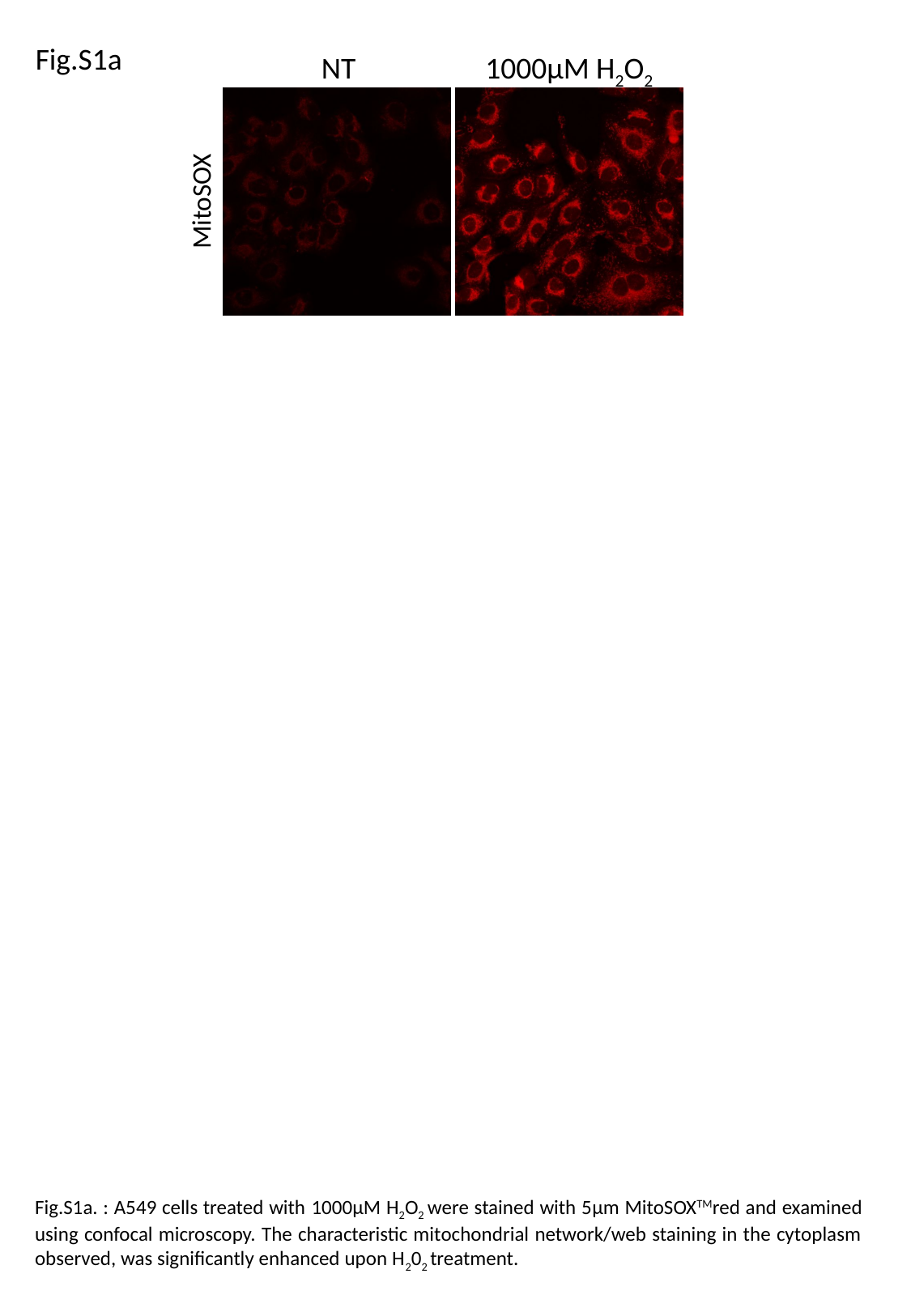

Fig.S1a
NT
1000μM H2O2
MitoSOX
Fig.S1a. : A549 cells treated with 1000μM H2O2 were stained with 5μm MitoSOXTMred and examined using confocal microscopy. The characteristic mitochondrial network/web staining in the cytoplasm observed, was significantly enhanced upon H202 treatment.

## Slide 2
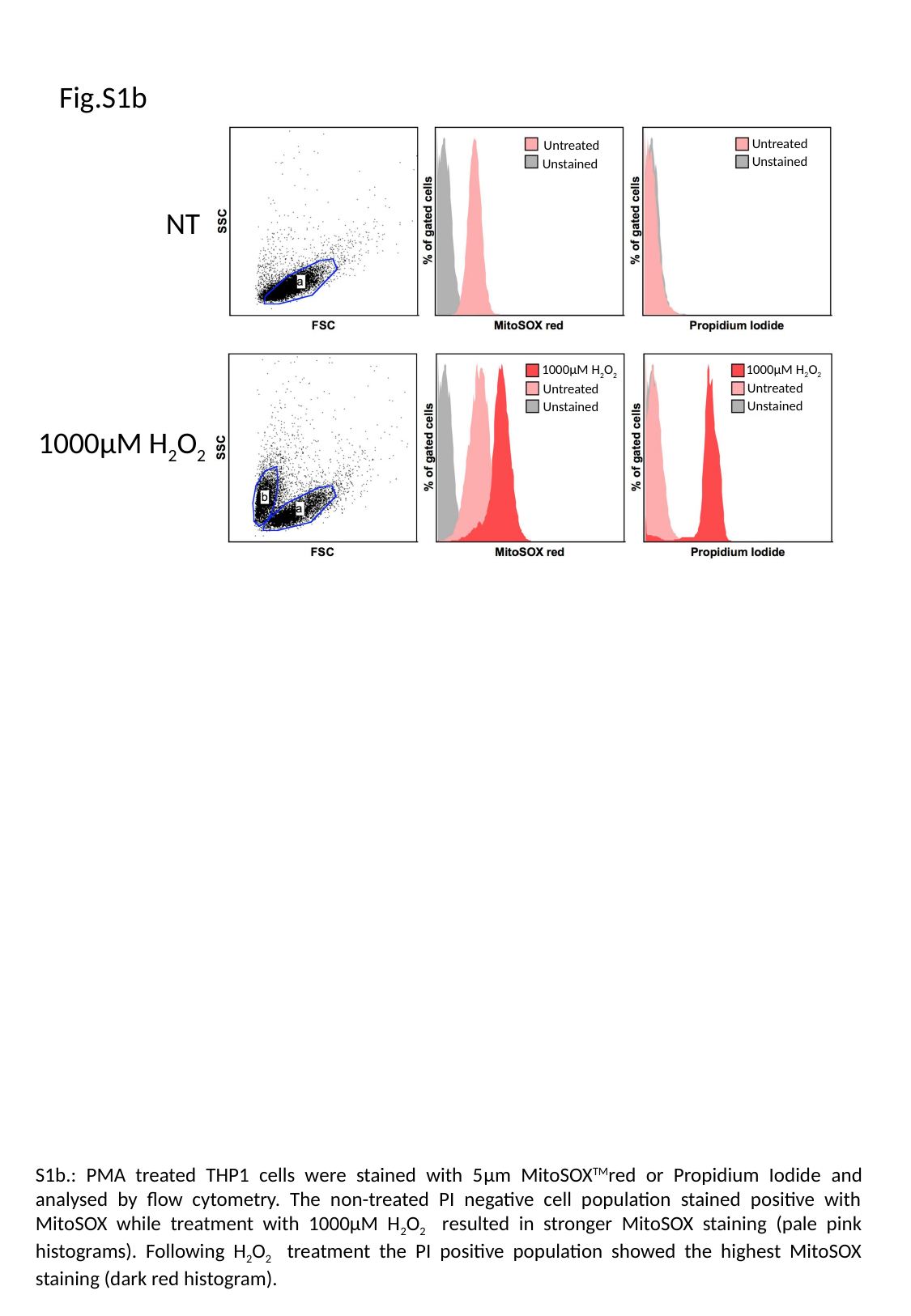

Fig.S1b
NT
1000μM H2O2
Untreated
Untreated
Unstained
Unstained
1000μM H2O2
1000μM H2O2
Untreated
Untreated
Unstained
Unstained
S1b.: PMA treated THP1 cells were stained with 5μm MitoSOXTMred or Propidium Iodide and analysed by flow cytometry. The non-treated PI negative cell population stained positive with MitoSOX while treatment with 1000μM H2O2 resulted in stronger MitoSOX staining (pale pink histograms). Following H2O2 treatment the PI positive population showed the highest MitoSOX staining (dark red histogram).

## Slide 3
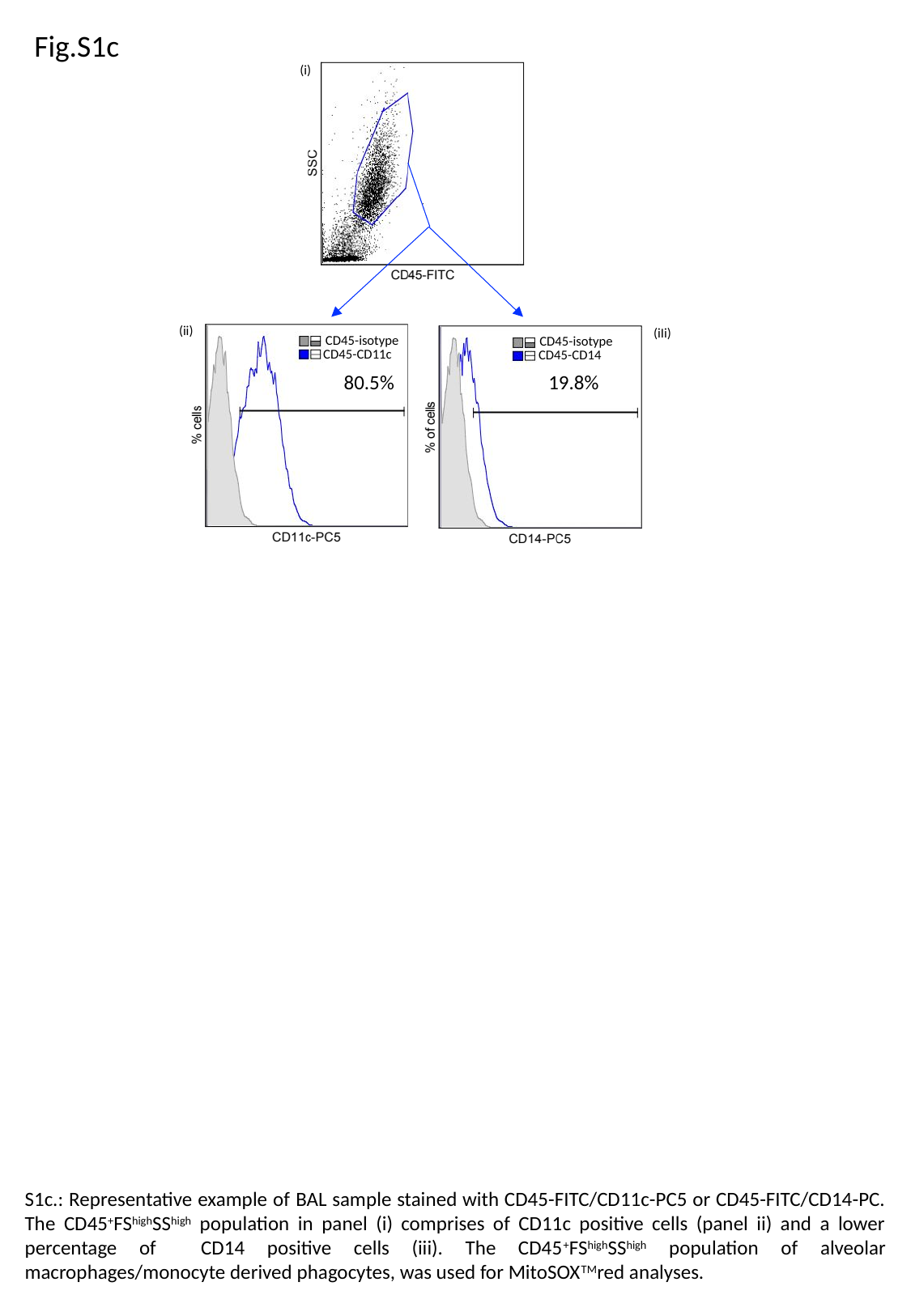

Fig.S1c
(i)
(ii)
(iIi)
CD45-isotype
CD45-isotype
CD45-CD11c
CD45-CD14
80.5%
19.8%
S1c.: Representative example of BAL sample stained with CD45-FITC/CD11c-PC5 or CD45-FITC/CD14-PC. The CD45+FShighSShigh population in panel (i) comprises of CD11c positive cells (panel ii) and a lower percentage of CD14 positive cells (iii). The CD45+FShighSShigh population of alveolar macrophages/monocyte derived phagocytes, was used for MitoSOXTMred analyses.

## Slide 4
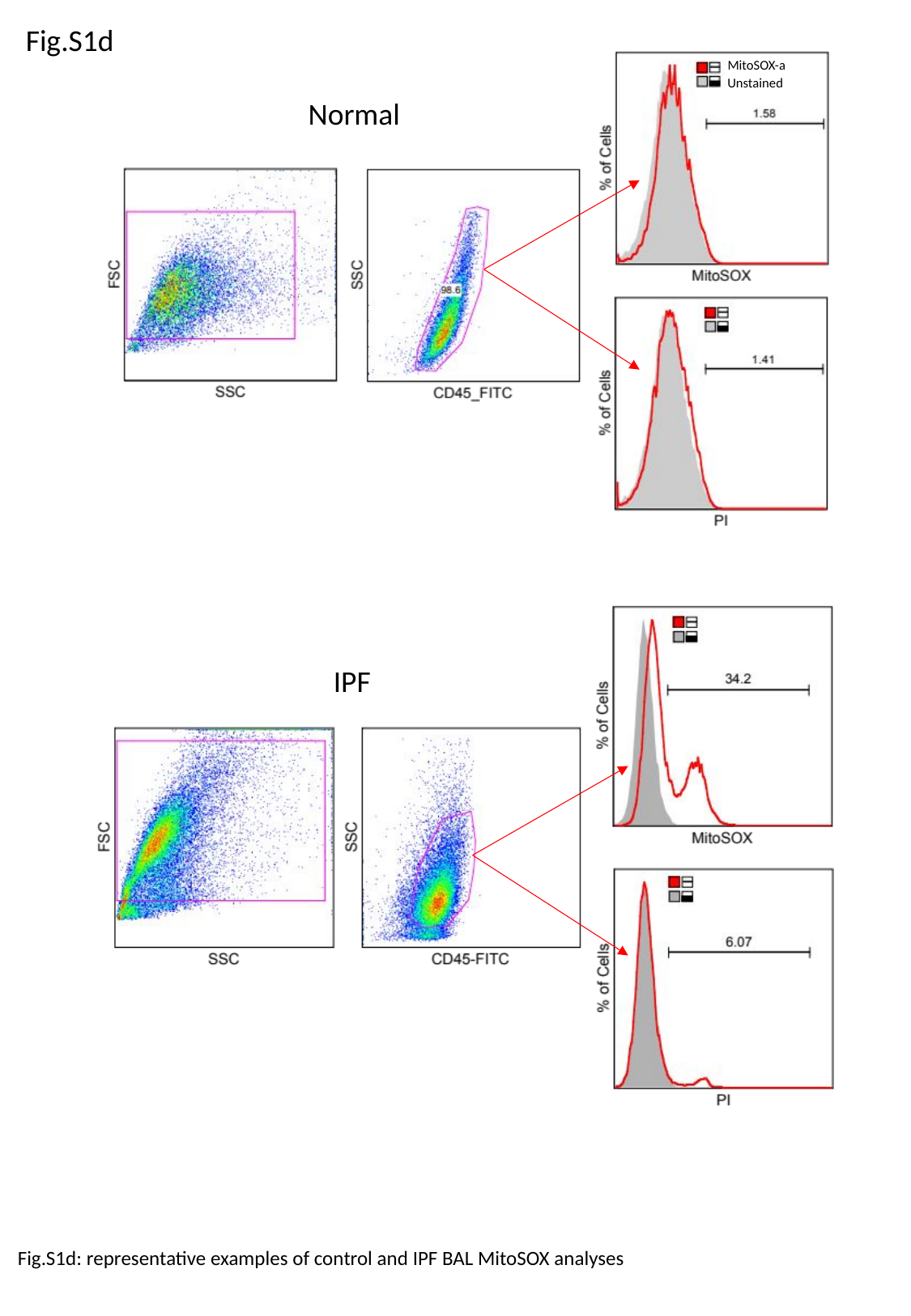

Fig.S1d
MitoSOX-a
Unstained
Normal
IPF
Fig.S1d: representative examples of control and IPF BAL MitoSOX analyses
