# Supplementary material for: Accumulation of damaged mitochondria in alveolar macrophages with reduced OXPHOS related gene expression in IPF
Source: Respir Res. 2019 Nov 27;20:264. doi: 10.1186/s12931-019-1196-6 (PMC6880424; doi:10.1186/s12931-019-1196-6)
Supplement: Supplementary file 2 — Additional file 2: Table S1. Primer sequences for qPCR. Table S2. Clinicopathological characteristics of the subjects included in Fig. 1b. Values are expressed as means±SD or medians with range. Table S3. Clinicopathological characteristics of the subjects included in Fig. 1f and g, Fig. 3c-d and Fig. 4a-d. Values are expressed as means±SD or medians with range. Table S4. Clinicopathological characteristics of the subjects included in Fig. 2b-d Values are expressed as means±SD or medians with range. Table S5. Clinicopathological characteristics of the subjects included in Fig. 3b. Values are expressed as means±SD or medians with range. [file 12931_2019_1196_MOESM2_ESM.docx]

**ONLINE DATA SUPPLEMENT**

**Accumulation of damaged mitochondria in alveolar macrophages with reduced OXPHOS related gene expression in IPF.**

Eliza Tsitoura^1^*, Eirini Vasarmidi ^1,2^*, Eleni Bibaki^1^, Athina Trachalaki^1,2^, Chara Koutoulaki^1^, George Papastratigakis^1^, Sevasti Papadogiorgaki^3^, George Chalepakis^3^, Nikos Tzanakis^1,2^ and Katerina M. Antoniou^1,2^.

* These authors contributed equally.

**Corresponding Author:**

Katerina M. Antoniou, MD, PhD

ERS Assembly 12 Secretary

Associate Professor in Respiratory Medicine,

Head of Molecular and Cellular Pneumonology Laboratory

Medical School, University of Crete

University Hospital of Heraklion, Crete, Greece

Tel: 0030 2810 392 433

Fax: 0030 2810 542650

Email address: [kantoniou@med.uoc.gr](mailto:kantoniou@med.uoc.gr)

**Supplementary Table 1.** Primer sequences for qPCR.

| **Gene name** | **Primer sequences** |
| --- | --- |
| PINK1 | F: ggagtatggagcagtcacttacag |
|  | R: ggcagcacatcagggtagtc |
| PARK2 | F: cacctacccagtgaccatga |
|  | R: cgacctccactgggaaac |
| NRF1 | F: ccatctggtggcctgaag |
|  | R: gtagtgcctgggtccatga |
| p62 | F: agctgccttgtacccacatc |
|  | R: cagagaagcccatggacag |
| BECLIN 1 | F: tcaccatccaggaactcaca |
|  | R: tggctcctctcctgagttagtc |
| HGB1 | F: gcttctgacacaactgtgttcactagc |
|  | R: caccaacttcatccacgttcacc |
| TGFb1 | F: actactacgccaaggaggtcac |
|  | R: tgcttgaacttgtcatagatttcg |
| Collagen1a1 | F: gggattccctggacctaaag |
|  | R: ggaacacctcgctctcca |
| GAPDH | F: agccacatcgctcagacac |
|  | R: gcccaatacgaccaaatcc |
| Mitochondria encoded genes |  |
| - For MT-DNA quantification |  |
| MT-ND1 (MRC complex I) | F: aacctctccacccttatcacaa |
|  | R: tcatattatggccaagggtca |
| MT-ND5 | F: tcttctcaccctaacaggtcaac |
|  | R: agggtggggttattttcgtt |
| - For mt-mRNA quantification |  |
| 12s rRNA/MT-RNR1 | F: tagaggagcctgttctgtaatcgat |
|  | R: cgacccttaagtttcataagggcta |
| MT-ND1(MRC complex I) | F: ccacctctagcctagccgttta |
|  | R: gggtcatgatggcaggagtaat |
| MT-ATP6 (MRC complex V) | F: tagccatacacaacactaaaggacga |
|  | R: gggcatttttaatcttagagcgaaa |

*PINK1 PTEN-induced kinase 1, PARK2 parkin E3 ubiquitin ligase , NRF1 nuclear respiratory factor 1, p62 selective autophagy receptor p62/sequestosome1 SQSTM1, BECN1* *Beclin-1* c*oiled-coil myosin-like BCL2-interacting protein, HGB1 haemoglobin 1 gene, TGFb1 transforming growth factor b1, GAPDH glyceraldehyde-3-phosphate dehydrogenase, MT mitochondrial, rRNA ribosomal RNA, MT-ND1 mitochondrial encoded NADH dehydrogenase 1, MT-ND5 mitochondrial encoded NADH dehydrogenase 5, MT-ATP6 ATP synthase 6, MRC mitochondria respiratory chain.*

**Supplementary Table 2.** Clinicopathological characteristics of the subjects included in Fig.1b. Values are expressed as means±SD or medians with range.

| Characteristics | Normal | IPF |
| --- | --- | --- |
| Number | 9 | 17 |
| Gender (Male/Female) | 8/1 | 13/4 |
| Packyears | 45.25(35.9) | 30.71(22) |
| Non smokers | 0 | 4 |
| Former smokers | 2 | 9 |
| Current smokers | 7 | 4 |
| Age (years) | 62.2±7.4 | 69.4±7 |
| Macrophages | 88.3±7 | 87.2±5.2 |
| Lymphocytes | 7.8±8 | 5.2±2.3 |
| Neutrophils | 3.1±3.7 | 5.6±4.6 |
| Eosinophils | 0.6±0.8 | 2.2±2.2 |
| FVC |  | 84.2±16.4 |
| FEV1 |  | 90.1±13.8 |
| FEV1/FVC |  | 85.3±5.7 |
| TLC |  | 78.4±13 |
| DLco |  | 58.7±13.3 |
| Kco |  | 91.3±21.1 |

**Supplementary Table 3.** Clinicopathological characteristics of the subjects included in Fig 1 f and g, Fig 3 c-d and Fig 4 a-d. Values are expressed as means±SD or medians with range.

| Characteristics | Normal | IPF |
| --- | --- | --- |
| Number | 14 | 42 |
| Gender (Male/Female) | 7/7 | 30/12 |
| Packyears | 40(2-100) | 18.5(0-90) |
| Non smokers | 0 | 17 |
| Former smokers | 3 | 20 |
| Current smokers | 11 | 5 |
| Age (years) | 61.6±7.4 | 73.1±6.8 |
| Macrophages | 90.7±3.9 | 81.2±12.2 |
| Lymphocytes | 6.1±2.7 | 9.4±10.9 |
| Neutrophils | 2.5±2.7 | 7.7±8.5 |
| Eosinophils | 0.4±0.5 | 1.6±1.7 |
| FVC |  | 85.7±21.2 |
| FEV1 |  | 93.6±18.9 |
| FEV1/FVC |  | 85.9±5.9 |
| TLC |  | 76.4±13.8 |
| DLco |  | 57.8±18.4 |
| Kco |  | 88.7±18.9 |

**Supplementary Table 4.** Clinicopathological characteristics of the subjects included in Fig. 2 b-d Values are expressed as means±SD or medians with range.

| Characteristics | Normal | IPF |
| --- | --- | --- |
| Number | 15 | 43 |
| Gender (Male/Female) | 9/6 | 32/11 |
| Packyears | 40(15-100) | 27(0-150) |
| Non smokers | 1 | 14 |
| Former smokers | 2 | 26 |
| Current smokers | 12 | 3 |
| Age (years) | 61.7±7.7 | 73.2±7.1 |
| Macrophages | 90.7±6.4 | 81.9±12.2 |
| Lymphocytes | 7.4±6.2 | 9.3±10.8 |
| Neutrophils | 1.25±1.2 | 7.1±8.5 |
| Eosinophils | 0.4±0.5 | 1.3±1.6 |
| FVC |  | 84.9±21.8 |
| FEV1 |  | 92.6±19.4 |
| FEV1/FVC |  | 85.4±5.6 |
| TLC |  | 74.1±13.6 |
| DLco |  | 55.7±17.7 |
| Kco |  | 86.9±19.1 |

**Supplementary Table 5.** Clinicopathological characteristics of the subjects included in Fig. 3b. Values are expressed as means±SD or medians with range.

| Characteristics | Normal | IPF |
| --- | --- | --- |
| Number | 9 | 24 |
| Gender (Male/Female) | 4/5 | 19/5 |
| Packyears | 40(10-100) | 27(0-150) |
| Non smokers | 0 | 7 |
| Former smokers | 1 | 16 |
| Current smokers | 8 | 1 |
| Age (years) | 63.3±6 | 72.7±7.9 |
| Macrophages | 91.6±3.7 | 81.6±11.5 |
| Lymphocytes | 6.3±3.7 | 7.4±8 |
| Neutrophils | 1.6±1.3 | 9±10.1 |
| Eosinophils | 0.4±0.5 | 1.6±2 |
| FVC |  | 80.1±15.8 |
| FEV1 |  | 89±14.7 |
| FEV1/FVC |  | 86.2±5.4 |
| TLC |  | 72.2±13.1 |
| DLco |  | 52.8±19.7 |
| Kco |  | 86.3±22.6 |
