# Supplementary material for: Accumulation of damaged mitochondria in alveolar macrophages with reduced OXPHOS related gene expression in IPF
Source: Respir Res. 2019 Nov 27;20:264. doi: 10.1186/s12931-019-1196-6 (PMC6880424; doi:10.1186/s12931-019-1196-6)
Supplement: Supplementary file 3 — Additional file 3: Figure S2. Mitochondrial DNA content assessed by mtDNA/gDNA ratio measuring both ND1(A) and ND5(B) relative to HGB1 in BAL cells. No statistical difference between control (n = 10) and IPF (n = 18). Representative western blot (C) and densitometry analysis (D) of mitochondria protein TOMM20 showing no statistical difference between control (n = 5) and IPF (n = 11). TOMM20 mean fluorescence intensity/cell measured from images acquired by confocal microscopy, per patient (E) and group (F). Non significant differences are highlighted by doted lines, all other comparisons showed statistically significant differences according to Kruskal Wallis test with Dunn’s test for multiple comparisons. (G) Representative image of TOMM20 staining of alveolar macrophages from IPF patient (p1). With the exception of (E) all for all other comparisons Mann-Whitney test was used, data are represented as median with interquartile ranges. [file 12931_2019_1196_MOESM3_ESM.pptx]

## Slide 1
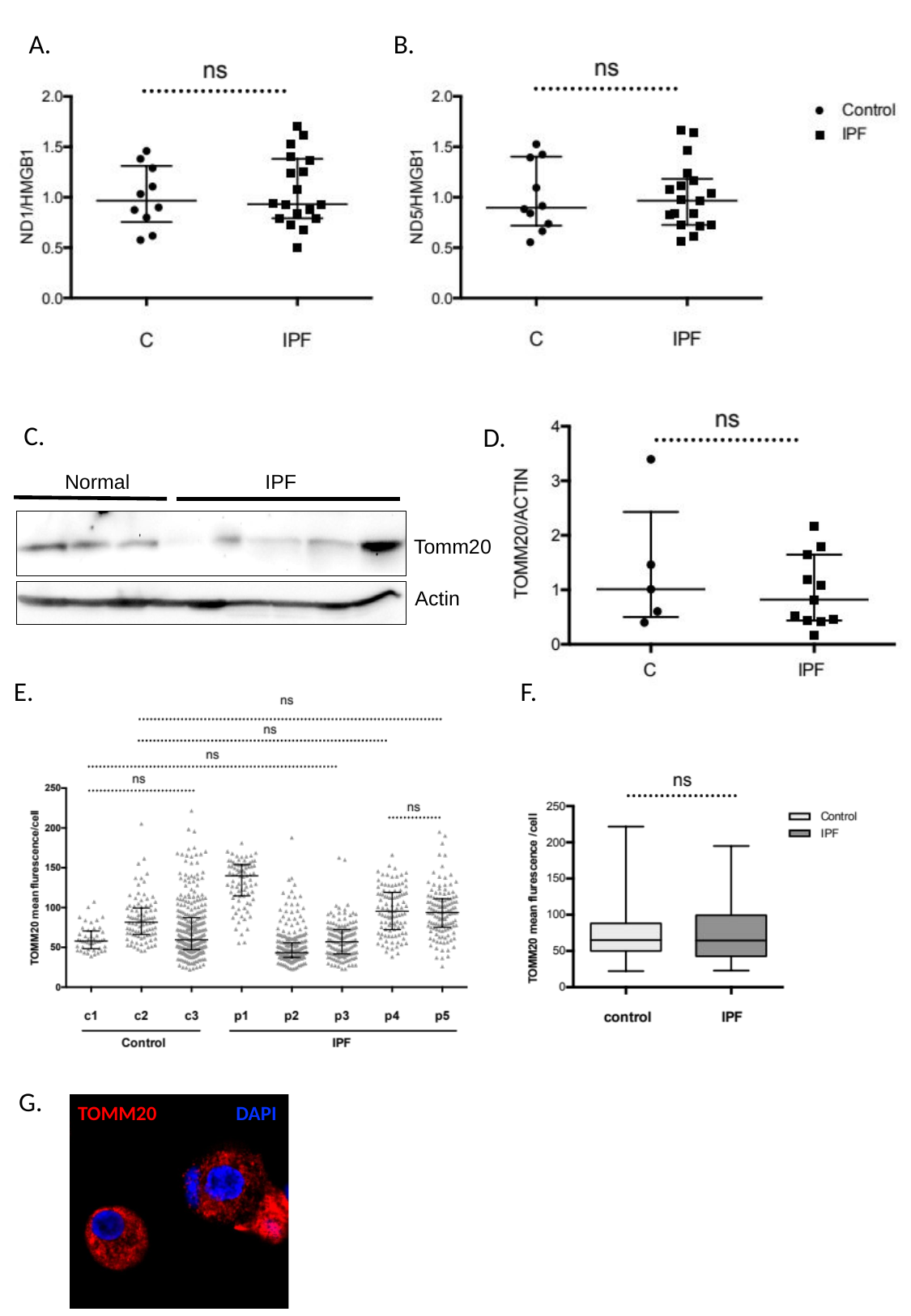

A.
B.
C.
D.
IPF
Normal
Tomm20
Actin
E.
F.
G.
TOMM20 DAPI

## Slide 2
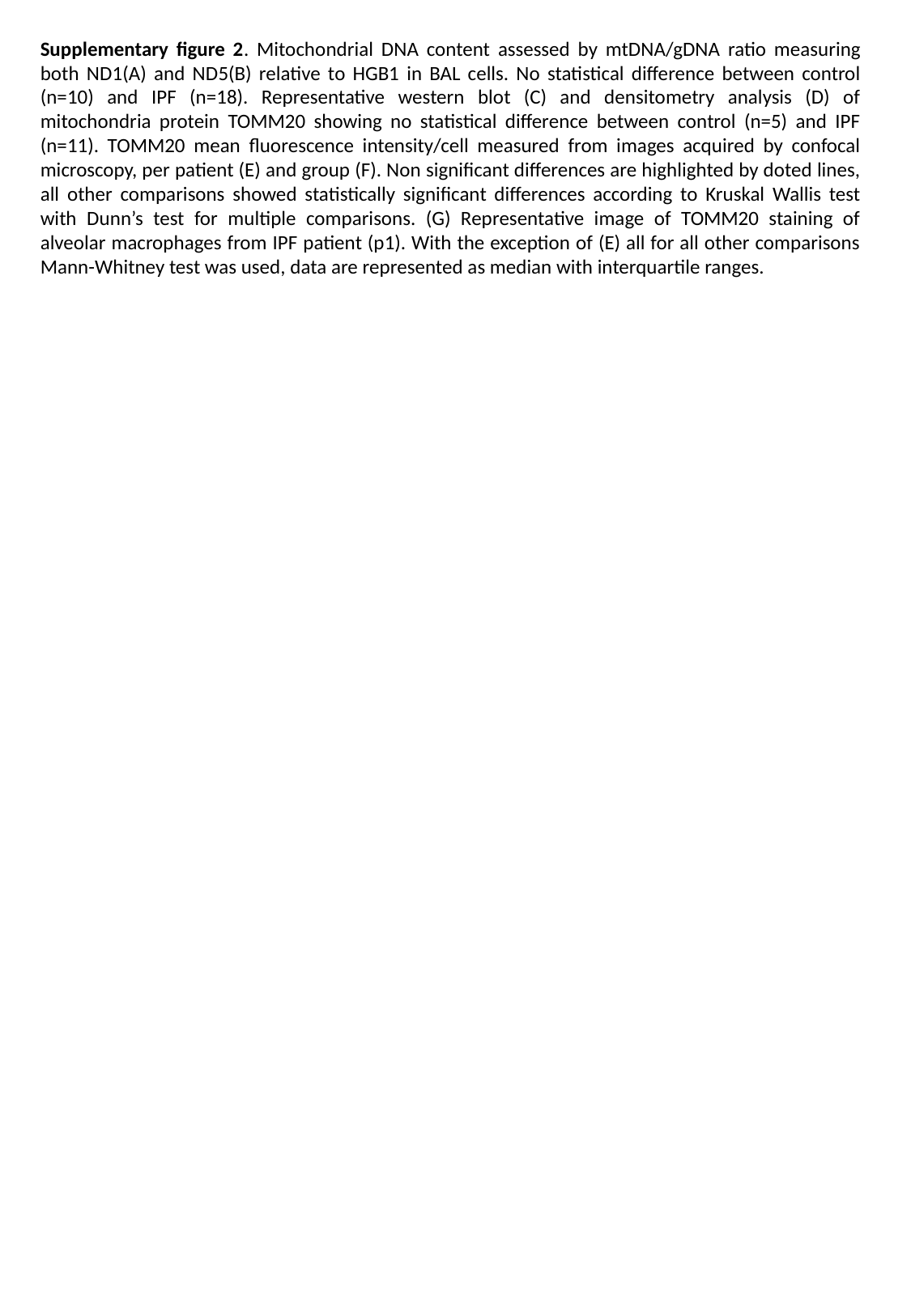

Supplementary figure 2. Mitochondrial DNA content assessed by mtDNA/gDNA ratio measuring both ND1(A) and ND5(B) relative to HGB1 in BAL cells. No statistical difference between control (n=10) and IPF (n=18). Representative western blot (C) and densitometry analysis (D) of mitochondria protein TOMM20 showing no statistical difference between control (n=5) and IPF (n=11). TOMM20 mean fluorescence intensity/cell measured from images acquired by confocal microscopy, per patient (E) and group (F). Non significant differences are highlighted by doted lines, all other comparisons showed statistically significant differences according to Kruskal Wallis test with Dunn’s test for multiple comparisons. (G) Representative image of TOMM20 staining of alveolar macrophages from IPF patient (p1). With the exception of (E) all for all other comparisons Mann-Whitney test was used, data are represented as median with interquartile ranges.
