# Supplementary material for: Accumulation of damaged mitochondria in alveolar macrophages with reduced OXPHOS related gene expression in IPF
Source: Respir Res. 2019 Nov 27;20:264. doi: 10.1186/s12931-019-1196-6 (PMC6880424; doi:10.1186/s12931-019-1196-6)
Supplement: Supplementary file 4 — Additional file 4: Figure S3. A, B: (i) LC3b I, (ii)II and (iii) LC3bII/I densitometry analyses from western blots performed on 2 independent IPF/control cohorts, C and D: relative mRNA expression of p62 and Beclin1. E. Representative images of LC3b immunostained alveolar macrophages from IPF and control samples treated with CQ or left untreated. All graphs represent median and interquartile ranges, no significant differences observed according to Mann-Whitney test. [file 12931_2019_1196_MOESM4_ESM.pptx]

## Slide 1
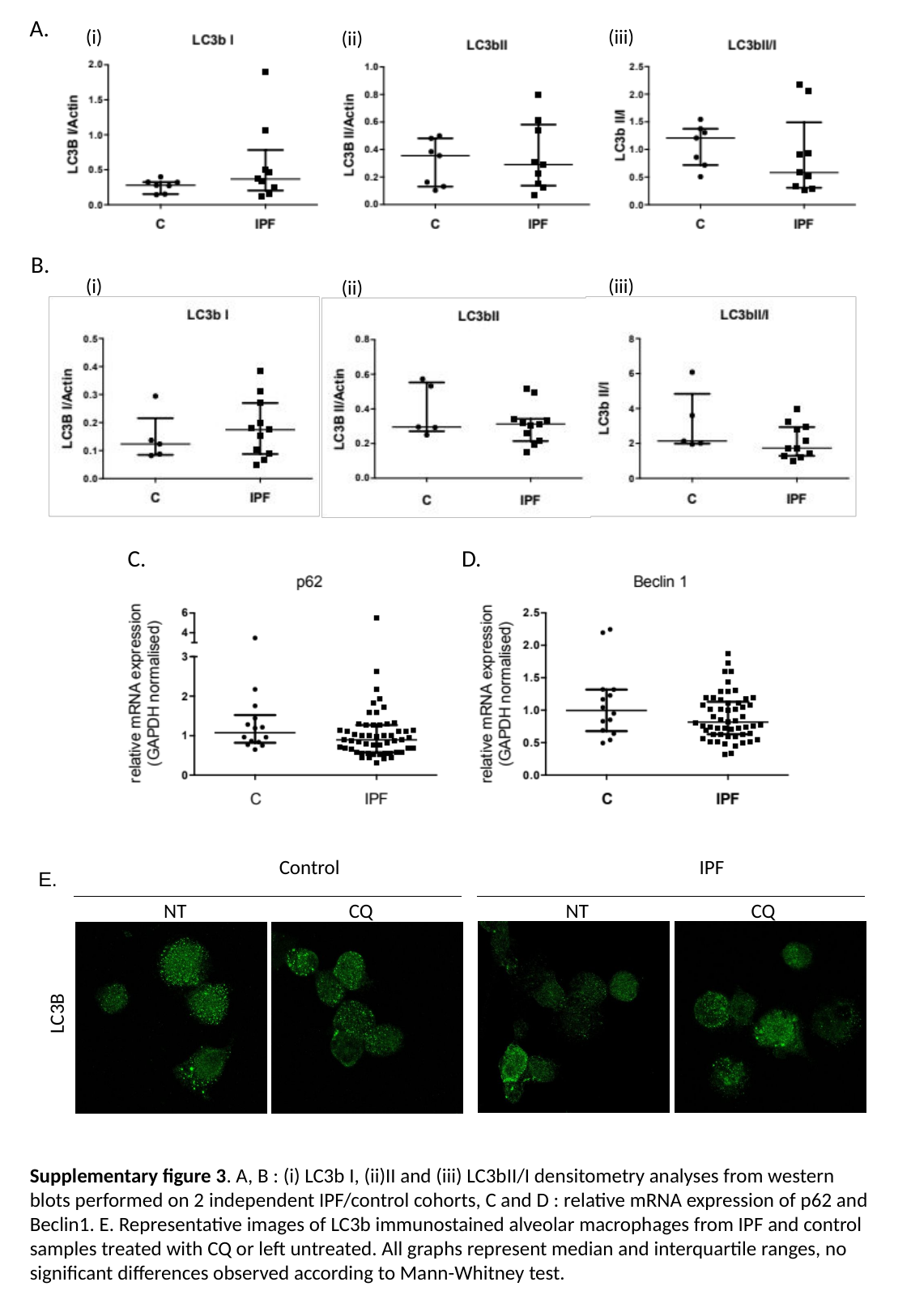

A.
(iii)
(i)
(ii)
B.
(iii)
(i)
(ii)
C.
D.
Control
IPF
E.
NT CQ
NT CQ
LC3B
Supplementary figure 3. A, B : (i) LC3b I, (ii)II and (iii) LC3bII/I densitometry analyses from western blots performed on 2 independent IPF/control cohorts, C and D : relative mRNA expression of p62 and Beclin1. E. Representative images of LC3b immunostained alveolar macrophages from IPF and control samples treated with CQ or left untreated. All graphs represent median and interquartile ranges, no significant differences observed according to Mann-Whitney test.
